# Supplementary material for: Facilitation of molecular motion to develop turn-on photoacoustic bioprobe for detecting nitric oxide in encephalitis
Source: Nat Commun. 2021 Feb 11;12:960. doi: 10.1038/s41467-021-21208-1 (PMC7878857; doi:10.1038/s41467-021-21208-1)
Supplement: Supplementary file 1 — Supplementary Information [file 41467_2021_21208_MOESM1_ESM.pdf]

Supplementary information

Facilitation of molecular motion to develop turn-on photoacoustic bioprobe for detecting nitric oxide in encephalitis

Qi *et al.*

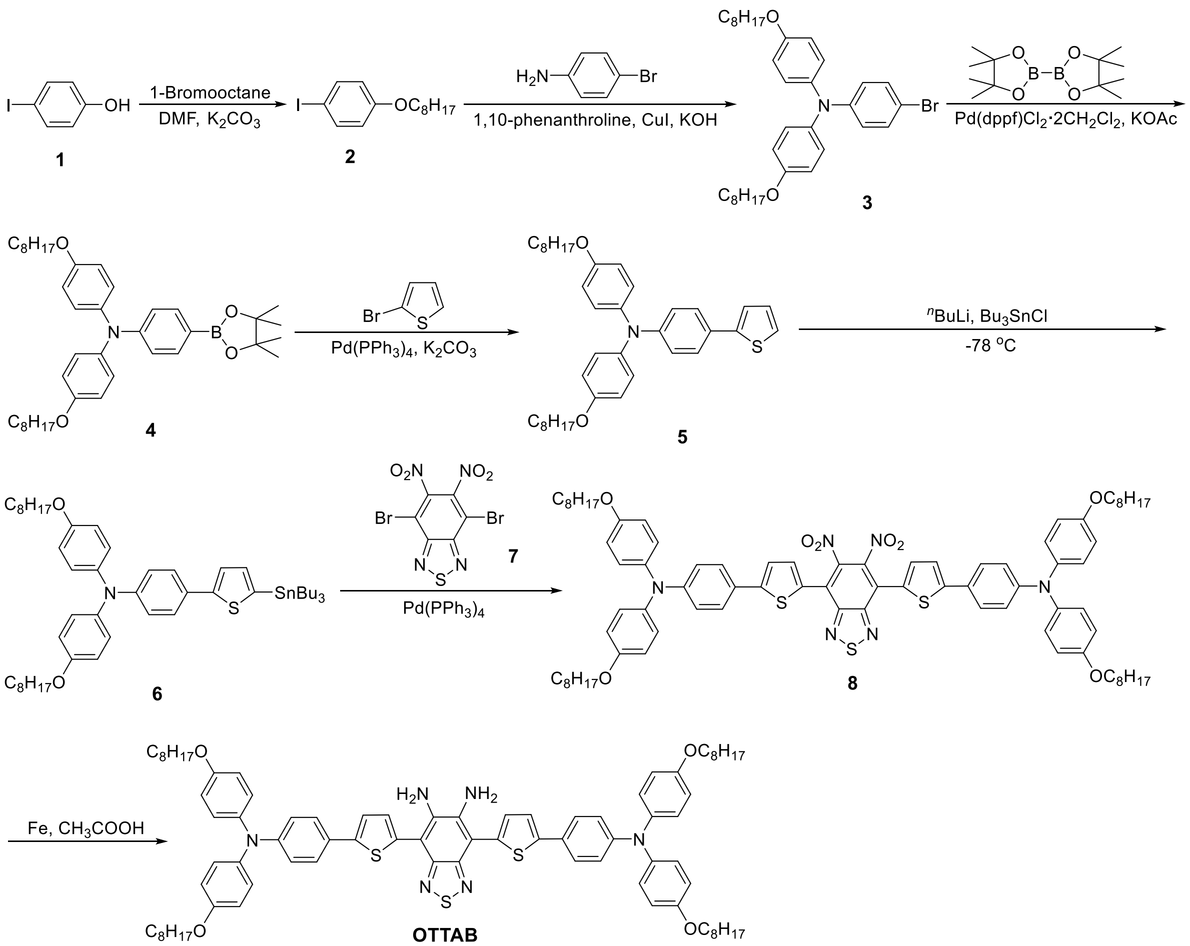

Supplementary Fig. 1 Synthetic route to OTTAB.

Syntheses processes and characterizations

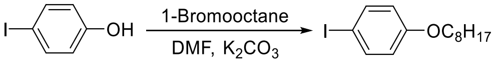

Synthesis of 1-iodo-4-(octyloxy)benzene (**2**)

The flask was first cleaned and blown with nitrogen, and dried in an oven. Before reaction, the flask was vacuumed and heated with a heat gun, then allowed to cool to room temperature in the vacuum state naturally. This process was repeated for three times to

remove residual air and water in the flask. 1-Bromooctane (4.24 g, 22 mmol), 4-iodophenol (4.4 g, 20 mmol), and K_2CO_3 (8.3 g, 60 mmol) were added into a 250 mL two-necked round-bottom flask, and the flask was vacuumed and purged with dry nitrogen three times. Then anhydrous DMF (120 mL) was added, and the mixture was heated to reflux and stirred for 24 h. After cooling down to room temperature, water was added, and the mixture was washed with CH_2Cl_2 three times. The organic phase was combined, dried with $MgSO_4$, and the solvent was evaporated under reduced pressure. The crude product was purified by column chromatography on silica gel using CH_2Cl_2/hexane (v/v 1:10) as the eluent to afford 1-iodo-4-(octyloxy)benzene as a colourless oil (83% yield). ^{1}H NMR (400 MHz, $CDCl_3$): δ 7.54 (d, 2H), 6.67 (d, 2H), 3.91 (t, 2H), 1.81–1.71 (m, 2H), 1.49–1.39 (m, 2H), 1.36–1.23 (m, 8H), 0.88 (t, 3H). ^{13}C NMR (100 MHz, $CDCl_3$): δ 159.03, 138.15, 116.94, 82.39, 68.14, 31.82, 29.34, 29.24, 29.16, 26.01, 22.67, 14.12.

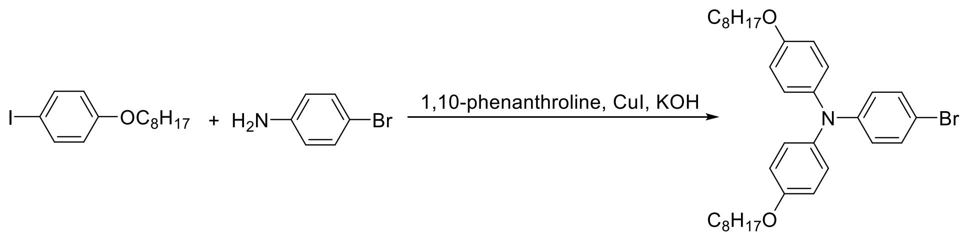

Synthesis of 4-bromo-*N*,*N*-bis(4-(octyloxy)phenyl)aniline (**3**)

4-Bromoaniline (1.03 g, 6 mmol), 1-iodo-4-(octyloxy)benzene (4.98 g, 15 mmol), 1,10-phenanthroline (0.18 g, 1 mmol), CuI (93 mg, 0.19 mmol), and KOH (5.04 g, 90 mmol) were added into a 250 mL two-necked round-bottom flask, which was vacuumed and purged with dry nitrogen three times. Then dry toluene (50 mL) was added into the flask, and the mixture was heated to reflux and stirred for 24 h. After cooling down to room

temperature, water was added, and the mixture was washed with CH_2Cl_2 three times. The organic phase was combined, dried with $MgSO_4$, and the solvent was evaporated under reduced pressure. The crude product was purified by column chromatography on silica gel using CH_2Cl_2/hexane (v/v 1:6) as the eluent to afford 4-bromo-*N*,*N*-bis(4-(octyloxy)phenyl)aniline as a viscous oil (76% yield). ^{1}H NMR (400 MHz, $CDCl_3$): δ 7.22 (d, 2H), 7.00 (d, 4H), 6.79 (t, 6H), 3.92 (t, 4H), 1.81–1.72 (m, 4H), 1.49–1.41 (m, 4H), 1.36–1.24 (m, 16H), 0.89 (t, 6H). ^{13}C NMR (100 MHz, $CDCl_3$): δ 155.65, 148.01, 140.36, 131.71, 126.57, 121.86, 115.33, 112.17, 68.28, 31.82, 29.37, 29.34, 29.25, 26.08, 22.66, 14.11.

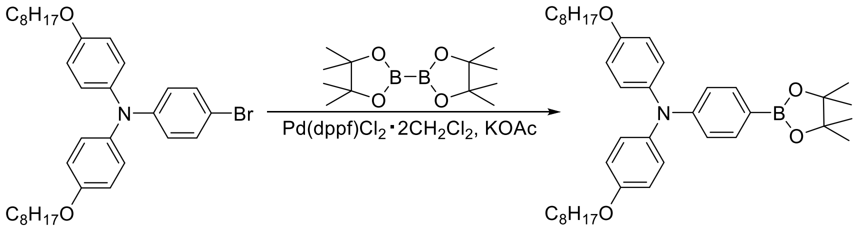

Synthesis of 4-(octyloxy)-*N*-(4-(octyloxy)phenyl)-*N*-(4-(4,4,5,5-tetramethyl-1,3,2-dioxaborolan-2-yl)phenyl)aniline (**4**)

4-Bromo-*N*,*N*-bis(4-(octyloxy)phenyl)aniline (2.32 g, 4 mmol), bis(pinacolato)diboron (1.14 g, 6 mmol), $Pd(dppf)Cl_2 \cdot 2CH_2Cl_2$ (147 mg, 0.2 mmol) and KOAc (1.18 g, 12 mmol) were added into a 100 mL two-necked round-bottom flask. The flask was vacuumed and purged with dry nitrogen three times. Then anhydrous 1,4-dioxane (30 mL) was added, and the mixture was heated to reflux and stirred for 24 h. The reaction mixture was cooled down to room temperature, and water was added, followed by extraction with CH_2Cl_2 three times. The organic phase was combined, and dried with $MgSO_4$. After removal of

the solvent under reduced pressure, the crude product was purified by column chromatography on silica gel using CH_2Cl_2/hexane (v/v 2:1) as the eluent to afford 4-(octyloxy)-*N*-(4-(octyloxy)phenyl)-*N*-(4-(4,4,5,5-tetramethyl-1,3,2-dioxaborolan-2-yl)phenyl)aniline as a light yellow solid (90% yield). ^{1}H NMR (400 MHz, $CDCl_3$): δ 7.59 (d, 2H), 7.04 (d, 4H), 6.86 (d, 2H), 6.81 (d, 4H), 3.92 (t, 4H), 1.82–1.71 (m, 4H), 1.52–1.41 (m, 4H), 1.41–1.31 (m, 20H), 1.29 (s, 12H), 0.89 (t, 6H). ^{13}C NMR (100 MHz, $CDCl_3$): δ 155.79, 151.45, 140.19, 135.73, 127.13, 118.52, 115.24, 83.38, 68.24, 31.83, 29.39, 29.36, 29.26, 26.09, 24.84, 22.68, 14.12.

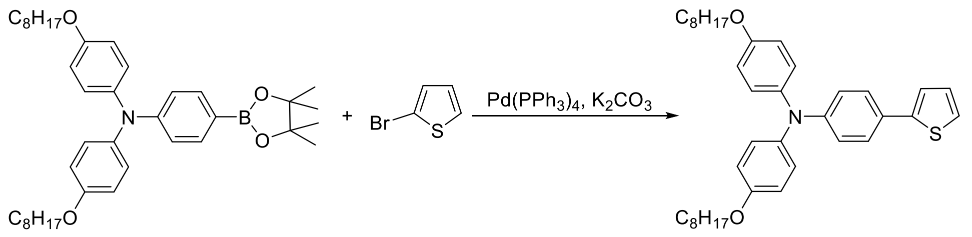

Synthesis of 4-(octyloxy)-*N*-(4-(octyloxy)phenyl)-*N*-(4-(thiophen-2-yl)phenyl)aniline (**5**)

4-(Octyloxy)-*N*-(4-(octyloxy)phenyl)-*N*-(4-(4,4,5,5-tetramethyl-1,3,2-dioxaborolan-2-yl)phenyl)aniline (2.2 g, 3.5 mmol), tributyl(thiophen-2-yl)stannane (0.57 g, 3.5 mmol), and $Pd(PPh_3)_4$ (58 mg, 0.05 mmol) were added into a 100 mL two-necked round-bottom flask. The flask was then vacuumed and purged with dry nitrogen three times, and anhydrous THF (40 mL) and aqueous K_2CO_3 solution (2 M, 10 mL) was added. The mixture was heated to reflux and stirred for 24 h. After cooling down to room temperature, water was added, and the mixture was washed with CH_2Cl_2 three times. The organic phase was combined, dried with $MgSO_4$, and the solvent was evaporated under reduced pressure. The crude product was purified by column chromatography on silica gel using

CH_2Cl_2/hexane (v/v 1:6) as the eluent to afford 4-(octyloxy)-*N*-(4-(octyloxy)phenyl)-*N*-(4-(thiophen-2-yl)phenyl)aniline as a light yellow solid (78% yield). ^{1}H NMR (400 MHz, $CDCl_3$): δ 7.39 (d, 4H), 7.17 (t, 4H), 7.10–6.98 (m, 10H), 6.91 (d, 4H), 6.86–6.77 (m, 8H), 3.92 (t, 8H), 1.84–1.71 (m, 8H), 1.51–1.40 (m, 8H), 1.40–1.21 (m, 32H), 0.89 (t, 12H). ^{13}C NMR (100 MHz, $CDCl_3$): δ 155.57, 148.29, 144.72, 140.55, 127.90, 126.63, 126.57, 126.50, 123.45, 121.67, 120.52, 115.31, 68.29, 31.86, 29.42, 29.40, 29.29, 26.13, 22.70, 14.15.

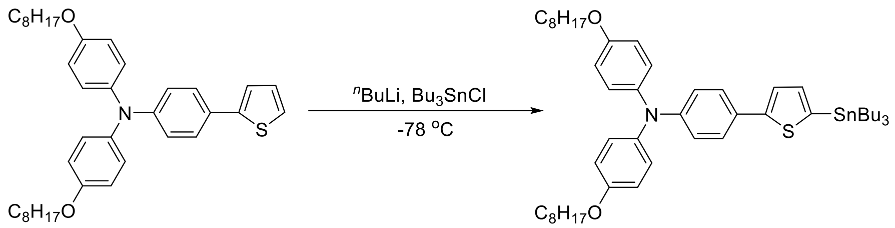

Synthesis of 4-(octyloxy)-*N*-(4-(octyloxy)phenyl)-*N*-(4-(5-(tributylstannyl)thiophen-2-yl)phenyl)aniline (**6**)

4-(Octyloxy)-*N*-(4-(octyloxy)phenyl)-*N*-(4-(thiophen-2-yl)phenyl)aniline (1.75 g, 3 mmol) was added into a 100 mL two-necked round-bottom flask. The flask was then vacuumed and purged with dry nitrogen three times, and anhydrous THF (40 mL) was added. Then the mixture was cooled with dry ice-acetone mixture to –78 ^oC, and maintained at this temperature for 15 min, followed by the addition of *n*-butyllithium (^{n}BuLi, 2.5 M hexane solution, 1.2 mL, 3 mmol). After stirring at this temperature for 2 h, tri-*n*-butyltin chloride (0.9 mL, 3.3 mmol) was added, and the mixture was slowly warmed to room temperature, and stirred overnight. Afterward, water was added to quench the reaction, and the mixture was extracted with CH_2Cl_2 three times. The organic phase was combined, dried with

$MgSO_4$, and the solvent was evaporated under reduced pressure. The crude product was used without further purification. ^1H NMR (400 MHz, $CDCl_3$): δ 7.39 (d, 4H), 7.17 (t, 4H), 7.10–6.98 (m, 10H), 6.91 (d, 4H), 6.86–6.77 (m, 8H), 3.92 (t, 8H), 1.84–1.71 (m, 8H), 1.51–1.40 (m, 8H), 1.40–1.21 (m, 32H), 0.89 (t, 12H). ^{13}C NMR (100 MHz, $CDCl_3$): δ 155.57, 148.29, 144.72, 140.55, 127.90, 126.63, 126.57, 126.50, 123.45, 121.67, 120.52, 115.31, 68.29, 31.86, 29.42, 29.40, 29.29, 26.13, 22.70, 14.15.

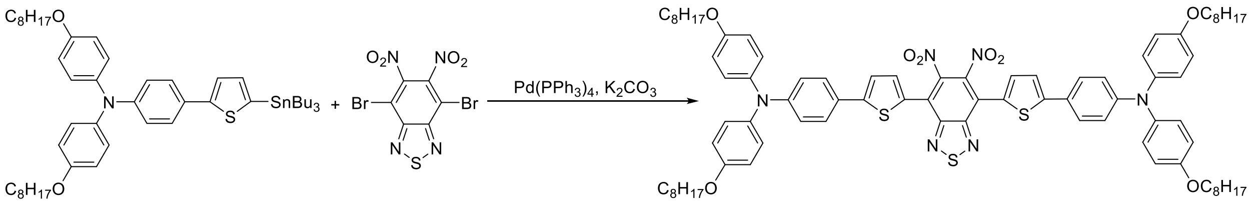

Synthesis of 4,4'-((5,6-dinitrobenzo[*c*][1,2,5]thiadiazole-4,7-diyl)bis(thiophene-5,2-diyl))bis(*N*,*N*-bis(4-(octyloxy)phenyl)aniline) (**7**)

4-(Octyloxy)-*N*-(4-(octyloxy)phenyl)-*N*-(4-(5-(tributylstannyl)thiophen-2-yl)phenyl)aniline (2.18 g, 2.5 mmol), 4,7-dibromo-5,6-dinitrobenzo[*c*][1,2,5]thiadiazole (422 mg, 1.1 mmol), and $Pd(PPh_3)_4$ (58 mg, 0.05 mmol) were added into a 100 mL two-necked round-bottom flask. The flask was then vacuumed and purged with dry nitrogen three times, and anhydrous THF (40 mL) was added. The mixture was heated to reflux and stirred for 24 h. After cooling down to room temperature, water was added, and the mixture was washed with CH_2Cl_2 three times. The organic phase was combined, dried with $MgSO_4$, and the solvent was evaporated under reduced pressure. The crude product was purified by column chromatography on silica gel using CH_2Cl_2/hexane (v/v 1:2) as the eluent to afford 4,4'-((5,6-dinitrobenzo[*c*][1,2,5]thiadiazole-4,7-diyl)bis(thiophene-5,2-diyl))bis(*N*,*N*-

bis(4-(octyloxy)phenyl)aniline) as a dark blue solid (78% yield). ^{1}H NMR (400 MHz, $CDCl_3$): δ 7.40 (d, 4H), 7.16 (d, 4H), 7.12–6.97 (m, 8H), 6.96–6.69 (m, 12H), 3.92 (t, 8H), 1.85–1.71 (m, 8H), 1.51–1.40 (m, 8H), 1.40–1.22 (m, 32H), 0.89 (t, 12H). ^{13}C NMR (100 MHz, $CDCl_3$): δ 159.94, 152.04, 151.63, 149.44, 141.16, 140.00, 132.19, 127.27, 127.03, 126.86, 124.61, 122.53, 120.21, 119.67, 115.38, 68.30, 31.84, 29.40, 29.36, 29.27, 26.11, 22.69, 14.13. HRMS (MALDI-TOF, *m*/*z*): $[M]^+$ calcd for $C_{82}H_{96}N_6O_8S_3$, 1388.6452; found, 1388.6475.

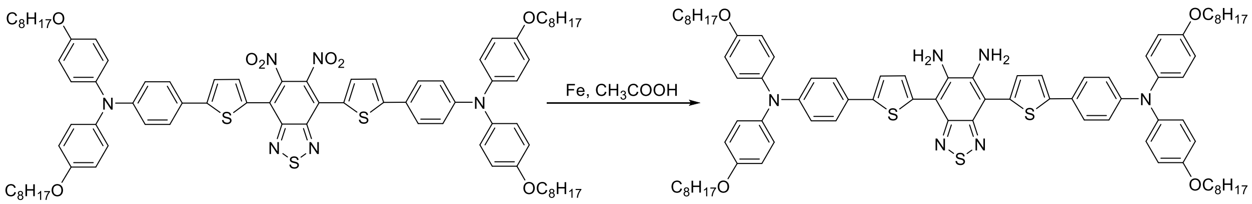

Synthesis of 4,7-bis(5-(4-(bis(4-(octyloxy)phenyl)amino)phenyl)thiophen-2-yl)benzo[*c*][1,2,5]thiadiazole-5,6-diamine (OTTAB)

To the mixture of 4,4'-((5,6-dinitrobenzo[*c*][1,2,5]thiadiazole-4,7-diyl)bis(thiophene-5,2-diyl))bis(*N*,*N*-bis(4-(octyloxy)phenyl)aniline) (0.613 g, 0.5 mmol) and acetic acid (80 mL) in a 250 mL two-necked round-bottom flask, iron powder (0.84 g, 15 mmol) was added. The mixture was heated to 80 °C, and stirred for 4 h. After cooling down to room temperature, water was added, and the mixture was washed with CH_2Cl_2 three times. The organic phase was combined, dried with $MgSO_4$, and the solvent was evaporated under reduced pressure. The crude product was purified by column chromatography on silica gel using CH_2Cl_2/hexane (v/v 2:1) as the eluent to afford 4,7-bis(5-(4-(bis(4-(octyloxy)phenyl)amino)phenyl)thiophen-2-yl)benzo[*c*][1,2,5]thiadiazole-5,6-diamine as a

dark red solid (71% yield). ^{1}H NMR (400 MHz, $CDCl_3$): δ 7.47 (4H), 7.31 (4H), 7.21–6.67 (20H), 4.51 (4H), 3.97 (8H), 1.82 (8H), 1.49 (8H), 1.34 (32H), 0.93 (12H). ^{13}C NMR (100 MHz, $CDCl_3$): δ 155.62, 150.79, 148.49, 146.30, 140.39, 139.24, 133.10, 129.69, 126.76, 126.57, 125.98, 121.91, 120.20, 115.30, 107.25, 68.28, 31.85, 29.41, 29.38, 29.28, 26.12, 22.70, 14.15. HRMS (MALDI-TOF, *m/z*): $[M]^+$ calcd for $C_{82}H_{100}N_6O_4S_3$, 1328.6968; found, 1328.6953.

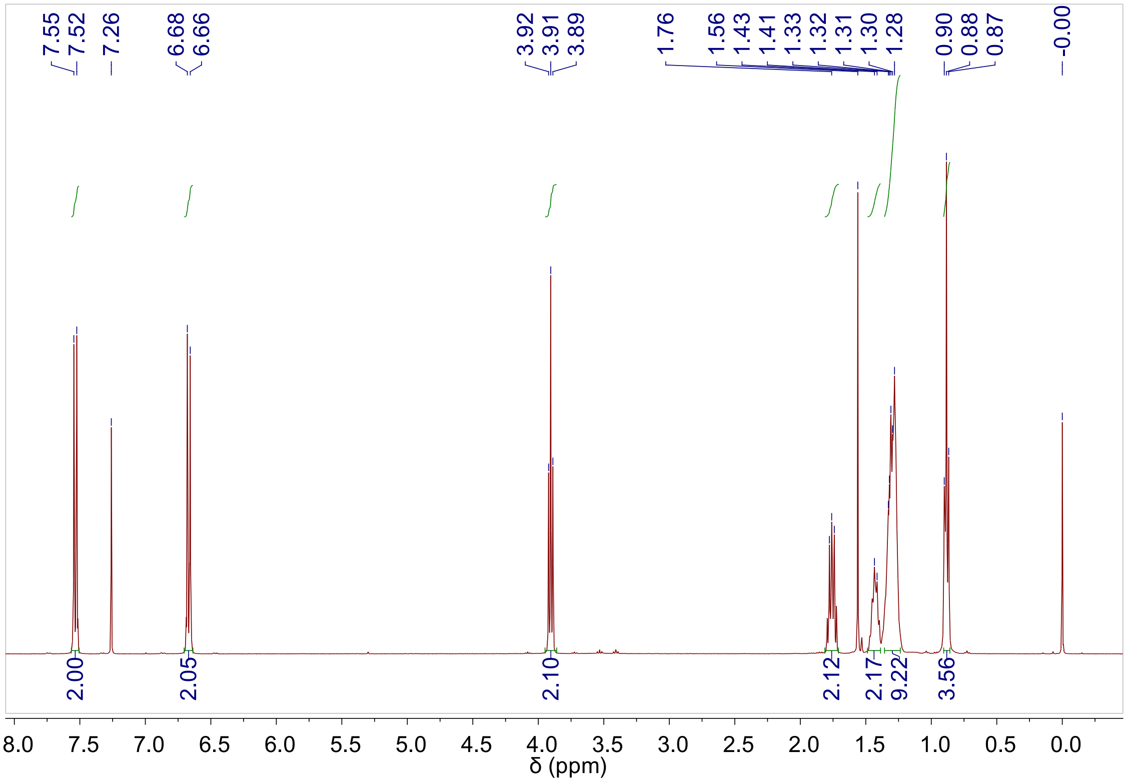

Supplementary Fig. 2 ^{1}H NMR spectrum of 1-iodo-4-(octyloxy)benzene in $CDCl_3$ at 298 K.

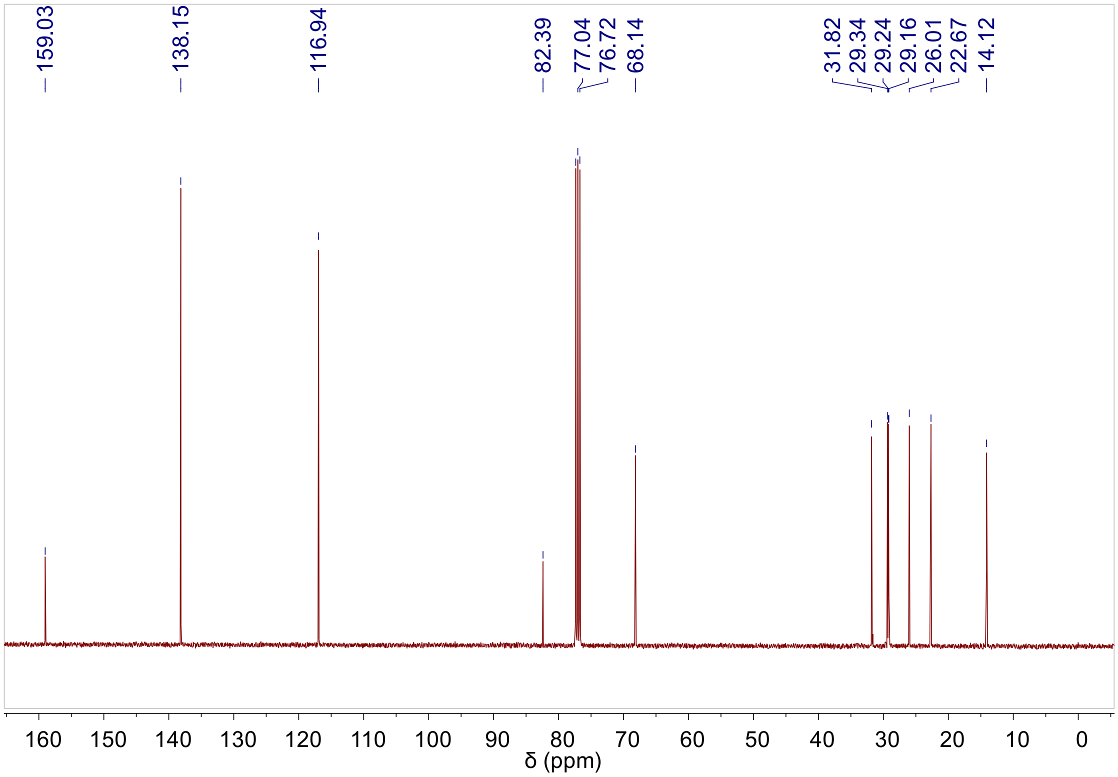

Supplementary Fig. 3 ^{13}C NMR spectrum of 1-iodo-4-(octyloxy)benzene in $CDCl_3$ at 298 K.

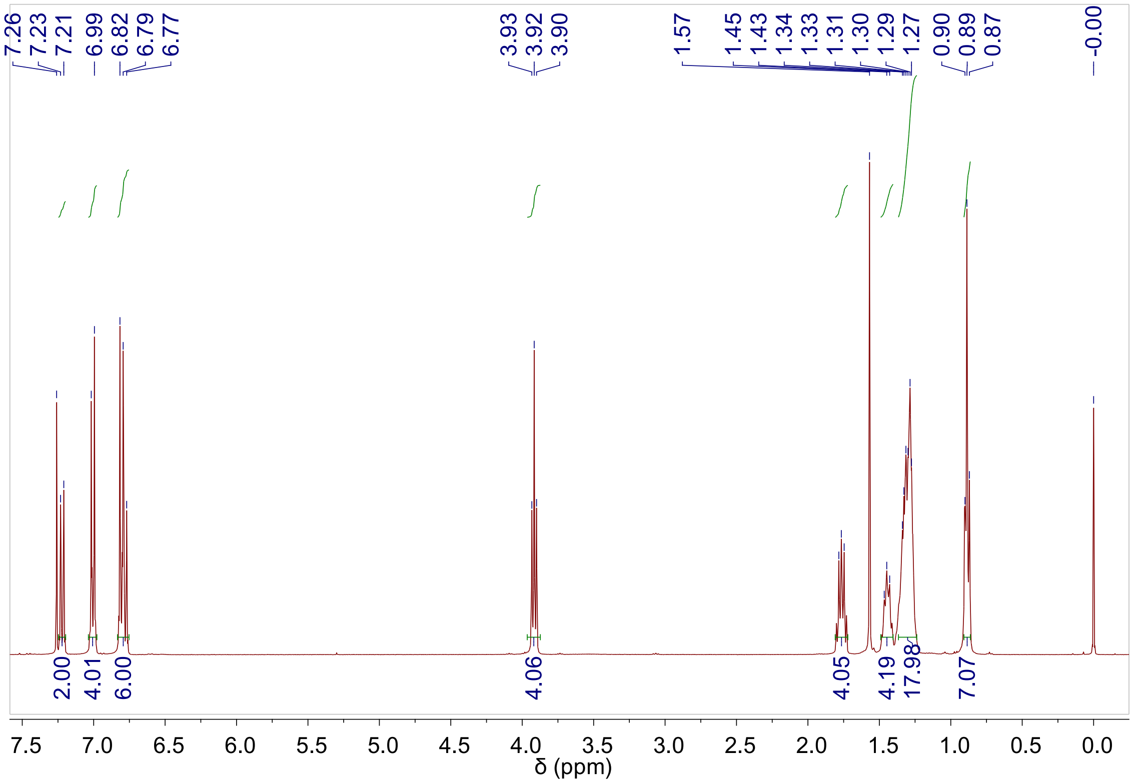

Supplementary Fig. 4 ^{1}H NMR spectrum of 4-bromo-*N*,*N*-bis(4-(octyloxy)phenyl)aniline in $CDCl_3$ at 298 K.

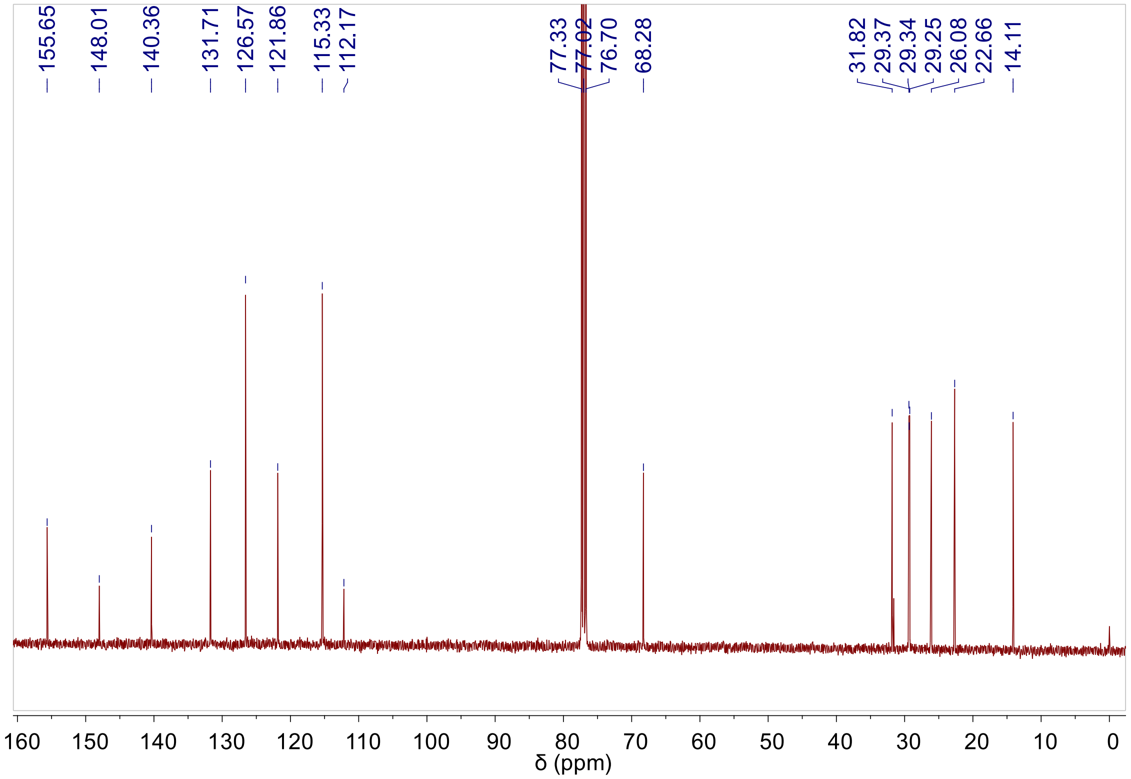

Supplementary Fig. 5 ^{13}C NMR spectrum of 4-bromo-*N*,*N*-bis(4-(octyloxy)phenyl)aniline in $CDCl_3$ at 298 K.

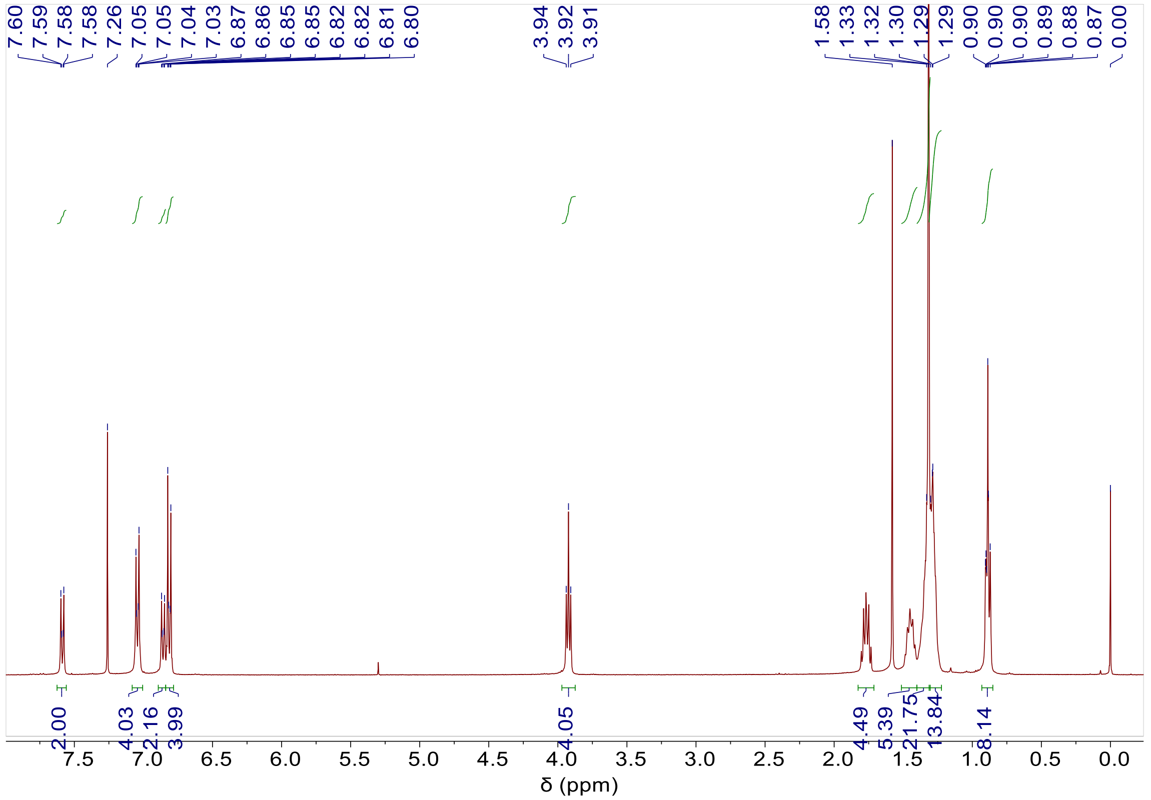

Supplementary Fig. 6 ^{1}H NMR spectrum of 4-(octyloxy)-*N*-(4-(octyloxy)phenyl)-*N*-(4-(4,4,5,5-tetramethyl-1,3,2-dioxaborolan-2-yl)phenyl)aniline in $CDCl_3$ at 298 K.

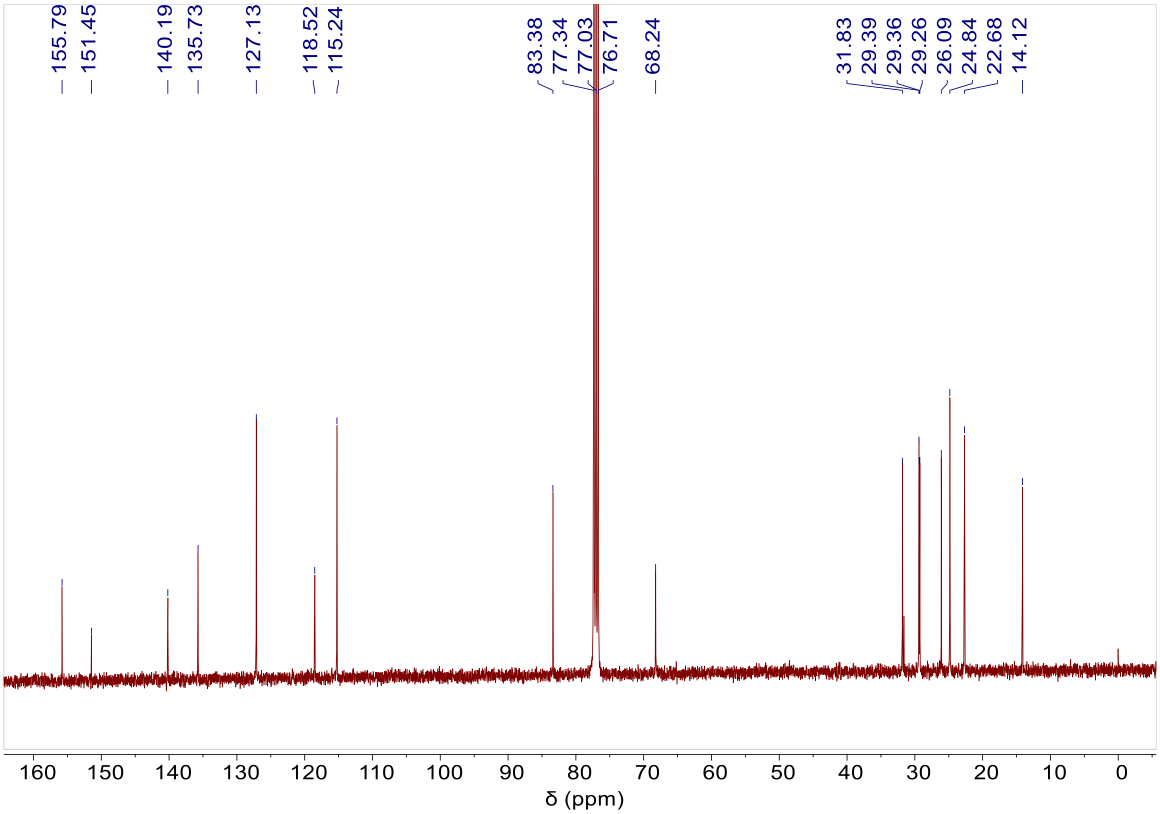

Supplementary Fig. 7 ^{13}C NMR spectrum of 4-(octyloxy)-*N*-(4-(octyloxy)phenyl)-*N*-(4-(4,4,5,5-tetramethyl-1,3,2-dioxaborolan-2-yl)phenyl)aniline in $CDCl_3$ at 298 K.

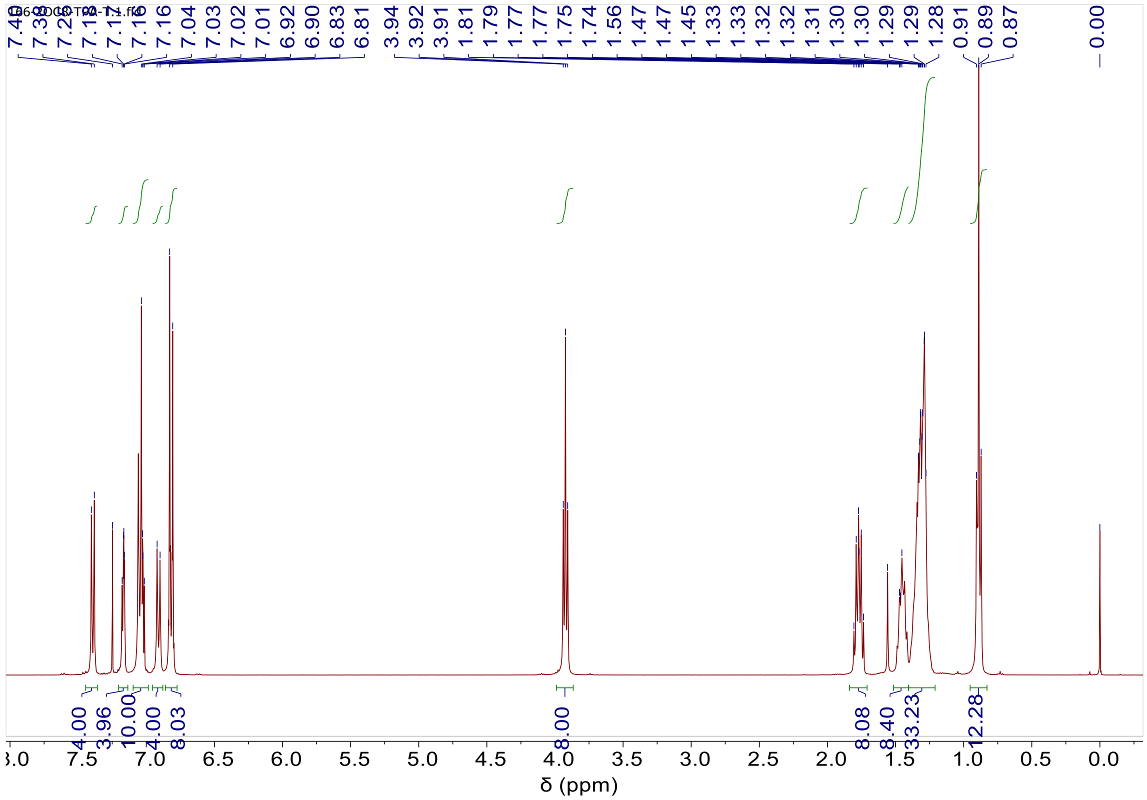

Supplementary Fig. 8 ^{1}H NMR spectrum of 4-(octyloxy)-*N*-(4-(octyloxy)phenyl)-*N*-(4-(thiophen-2-yl)phenyl)aniline in $CDCl_3$ at 298 K.

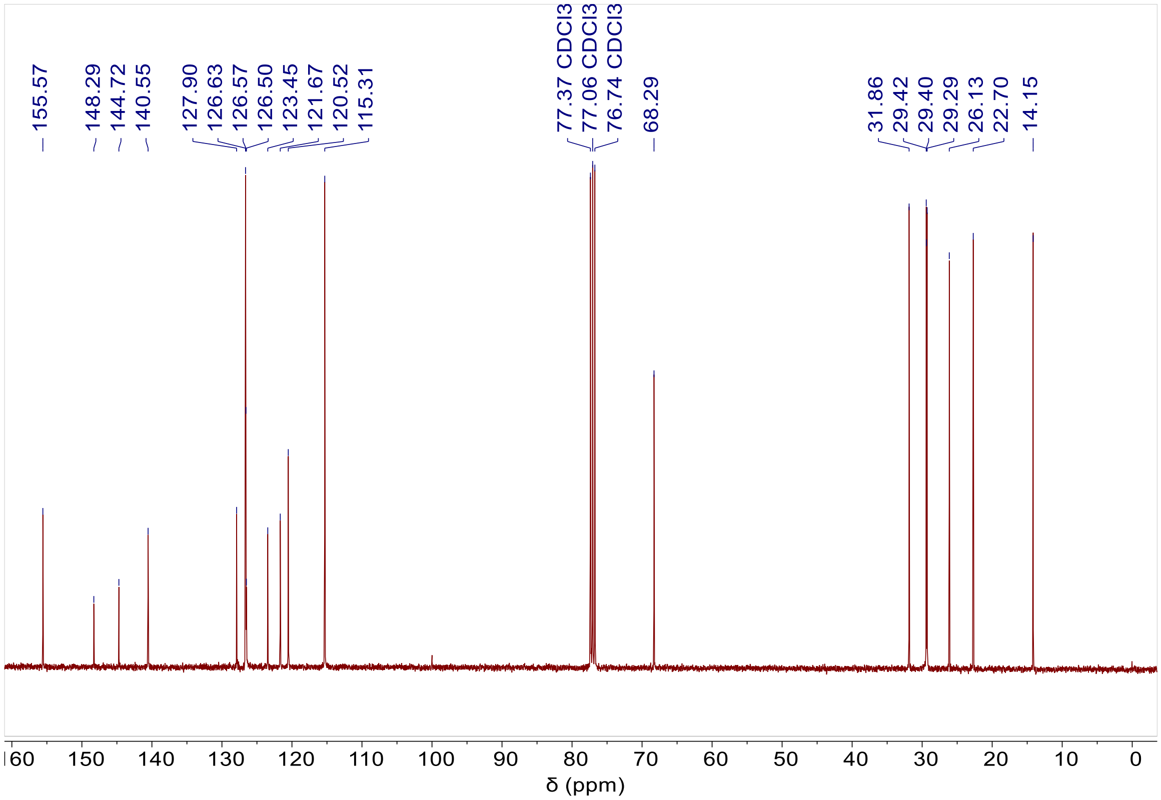

Supplementary Fig. 9 ^{13}C NMR spectrum of 4-(octyloxy)-*N*-(4-(octyloxy)phenyl)-*N*-(4-(thiophen-2-yl)phenyl)aniline in $CDCl_3$ at 298 K.

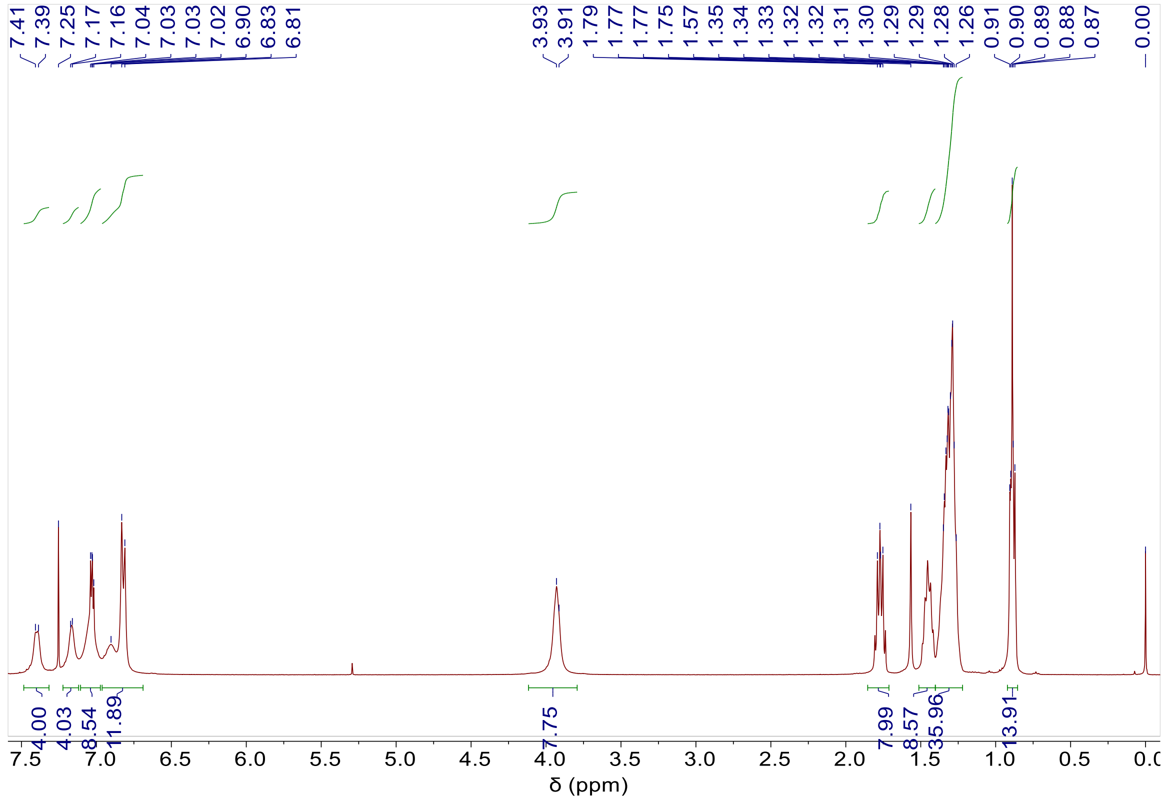

Supplementary Fig. 10 ^{1}H NMR spectrum of 4,4'-((5,6-dinitrobenzo[*c*][1,2,5]thiadiazole-4,7-diyl)bis(thiophene-5,2-diyl))bis(*N*,*N*-bis(4-(octyloxy)phenyl)aniline) in $CDCl_3$ at 298 K.

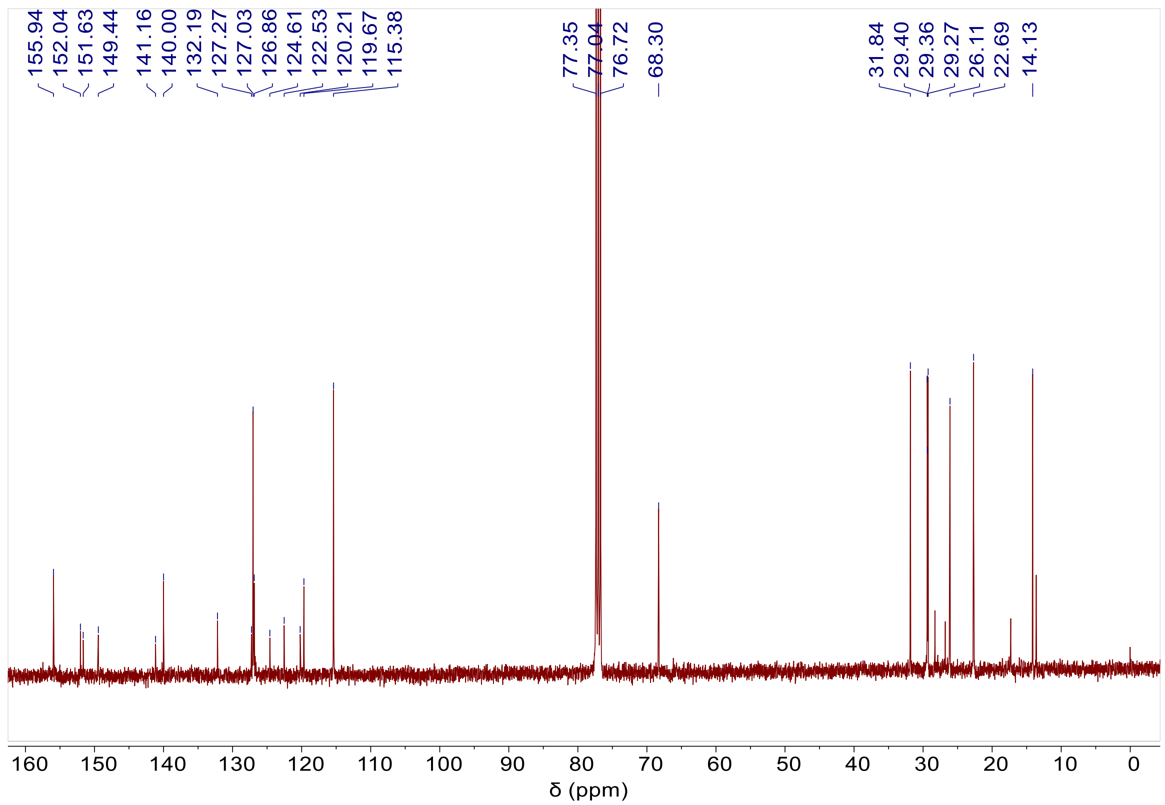

Supplementary Fig. 11 ^{13}C NMR spectrum of 4,4'-((5,6-dinitrobenzo[*c*][1,2,5]thiadiazole-4,7-diyl)bis(thiophene-5,2-diyl))bis(*N*,*N*-bis(4-(octyloxy)phenyl)aniline) in $CDCl_3$ at 298 K.

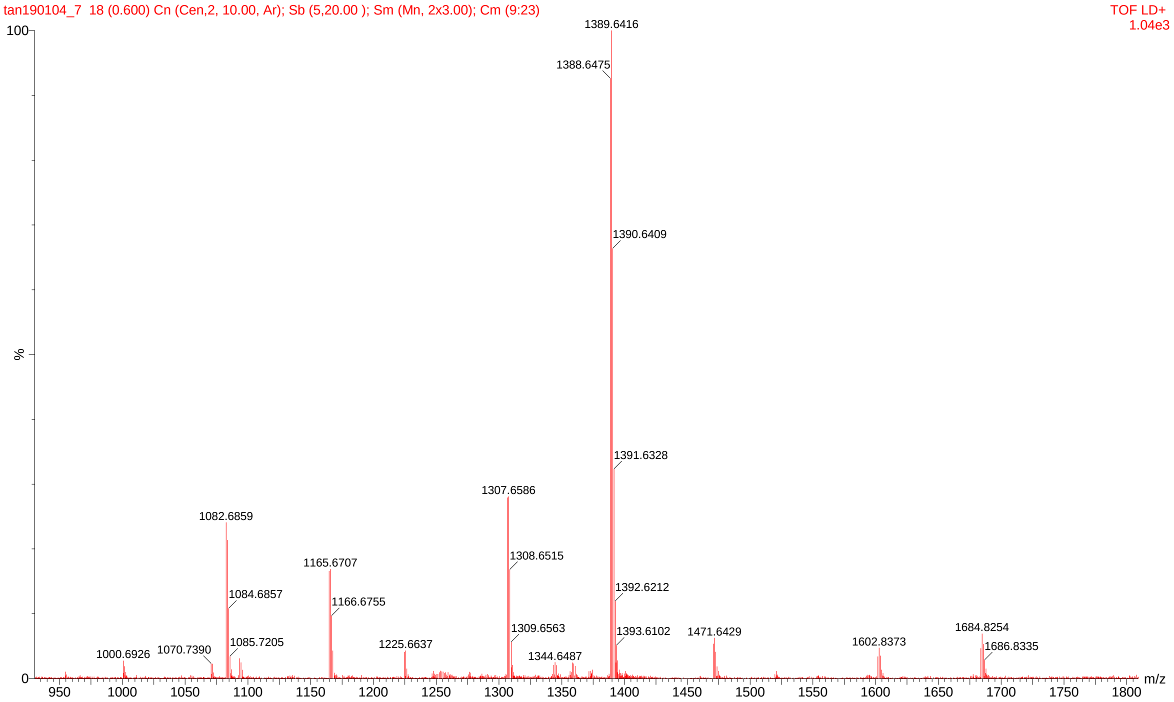

Supplementary Fig. 12 HRMS of 4,4'-((5,6-dinitrobenzo[*c*][1,2,5]thiadiazole-4,7-diyl)bis(thiophene-5,2-diyl))bis(*N*,*N*-bis(4-(octyloxy)phenyl)aniline).

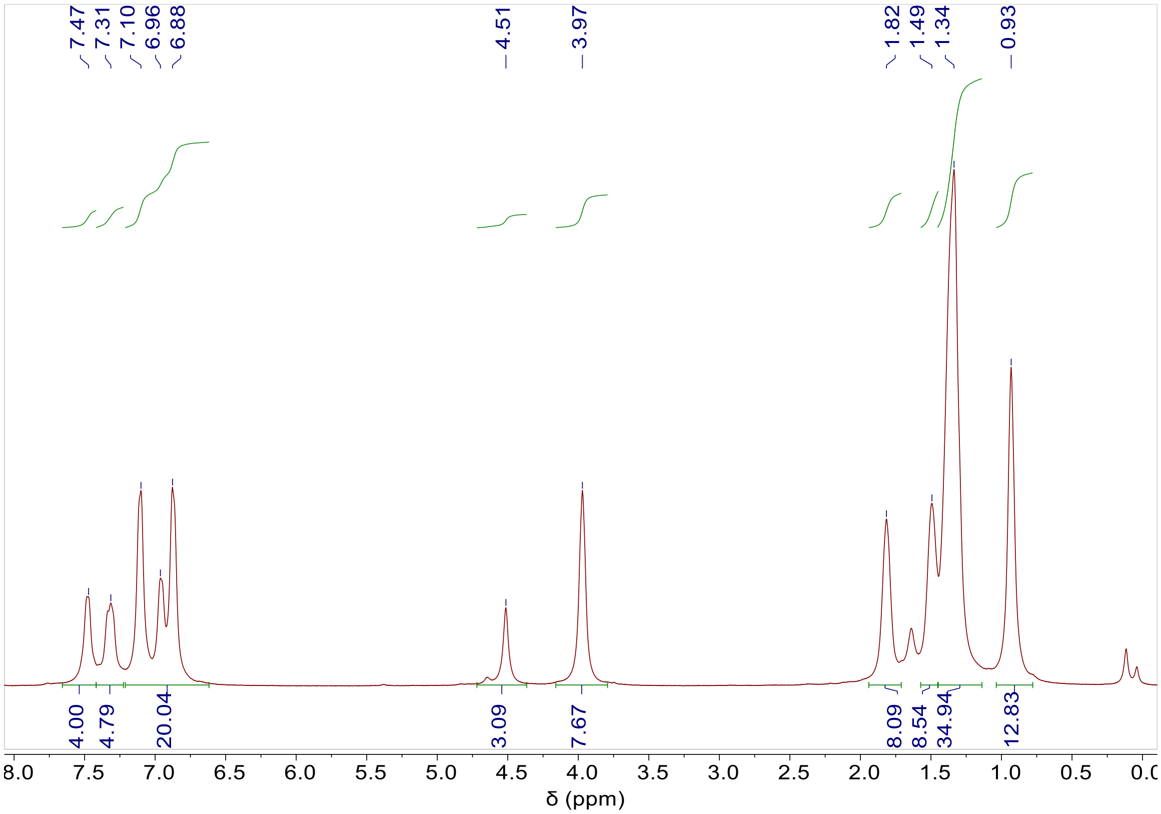

Supplementary Fig. 13 ^{1}H NMR spectrum of OTTAB in $CDCl_3$ at 298 K.

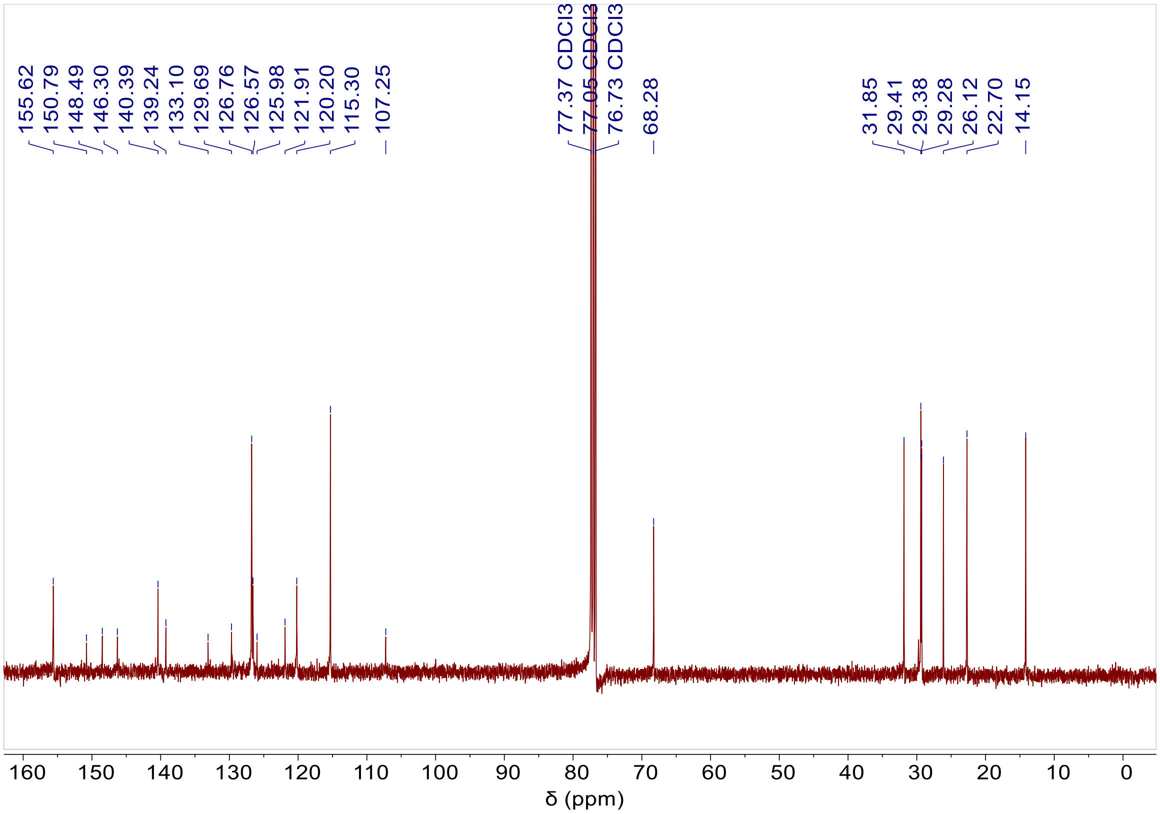

Supplementary Fig. 14 ^{13}C NMR spectrum of OTTAB in $CDCl_3$ at 298 K.

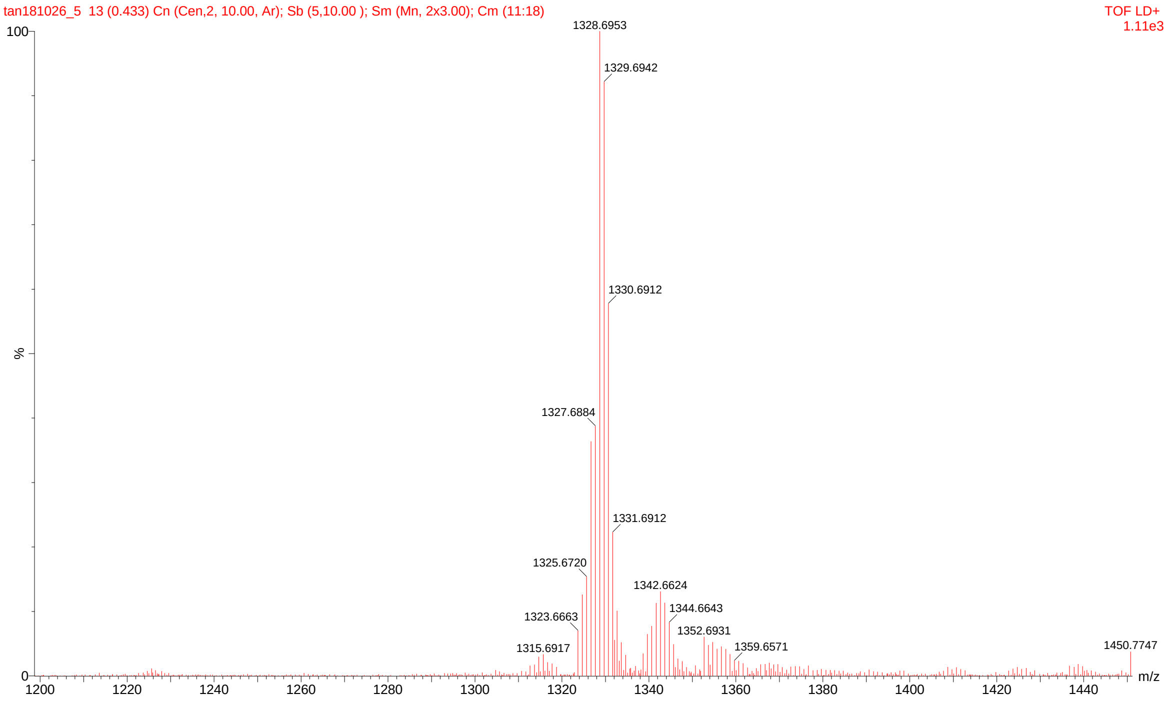

Supplementary Fig. 15 HRMS of OTTAB.

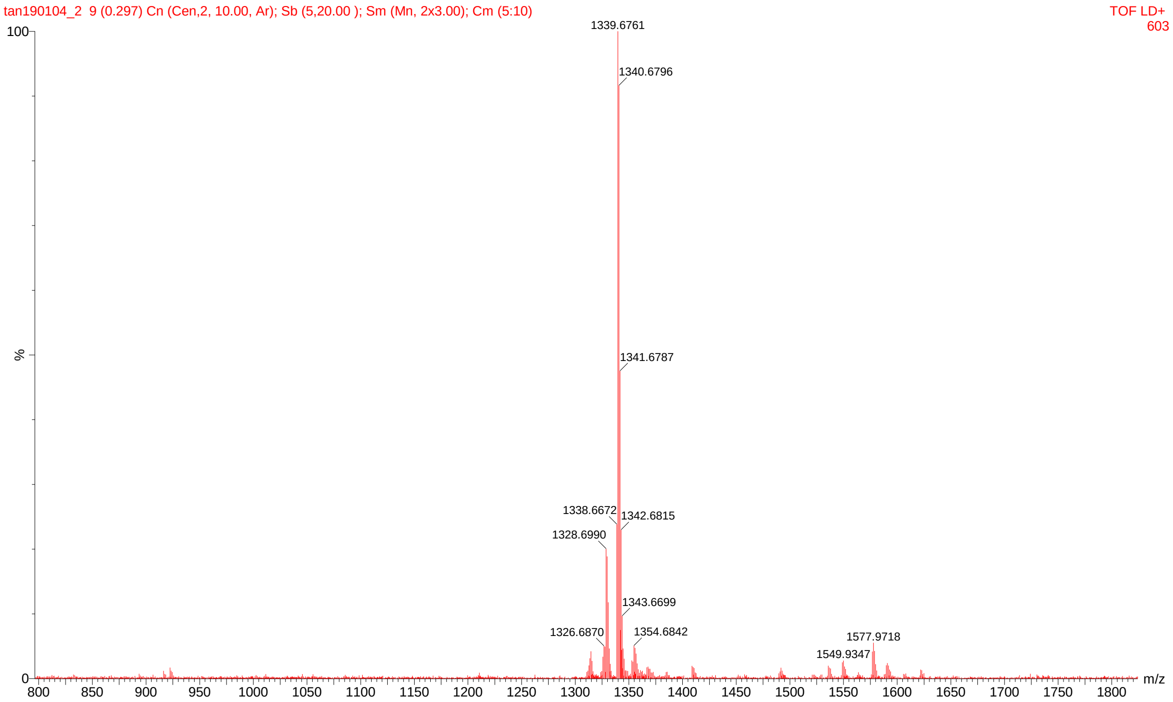

Supplementary Fig. 16 HRMS of OTTTB (HRMS (MALDI-TOF, *m/z*): $[M]^+$ calcd for $C_{82}H_{97}N_7O_4S_3$, 1339.6764; found, 1339.6761).

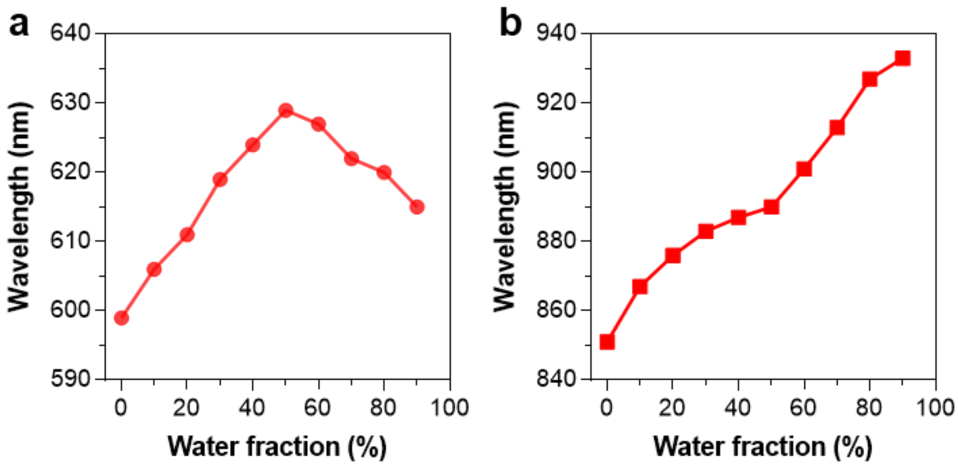

Supplementary Fig. 17 Plot of the maximal PL wavelength of **a** OTTAB and **b** OTTTB versus water fractions in THF/water mixture. I_0 and I are the PL peak intensities in pure THF (f_w = 0) and THF/water mixtures with specific water fractions, respectively.

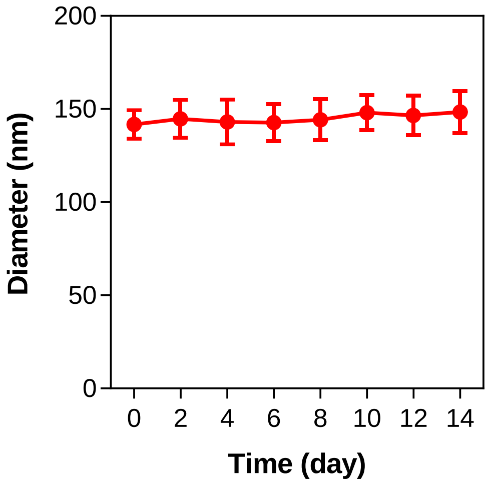

Supplementary Fig. 18 Average diameters of OTTAB NPs in PBS after storing in ambient condition for different time. Data are presented as mean ± s.d. (n = 3 independent experiments).

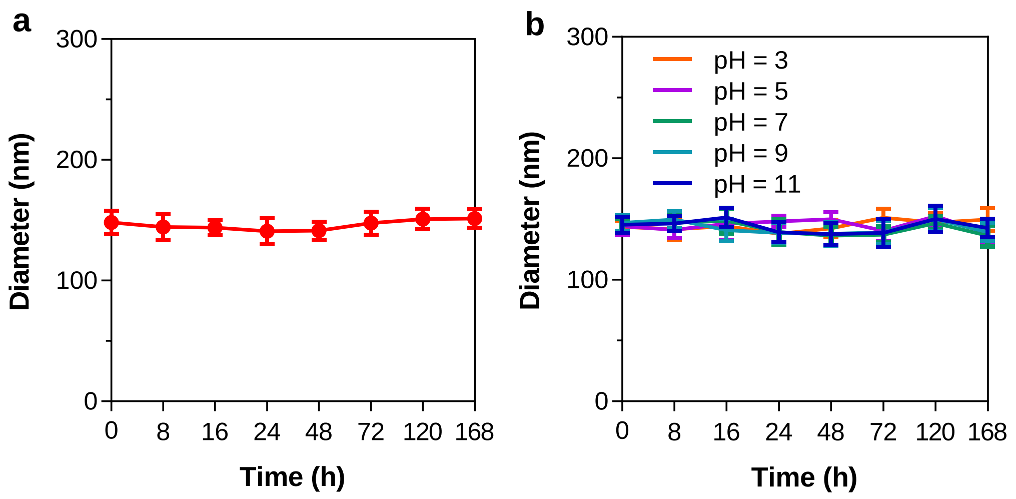

Supplementary Fig. 19 Average diameters of OTTAB NPs in different conditions: **a** Dulbecco's Modified Eagle's Medium (DMEM) and **b** different pH environments. Data are presented as mean ± s.d. (*n* = 3 independent experiments).

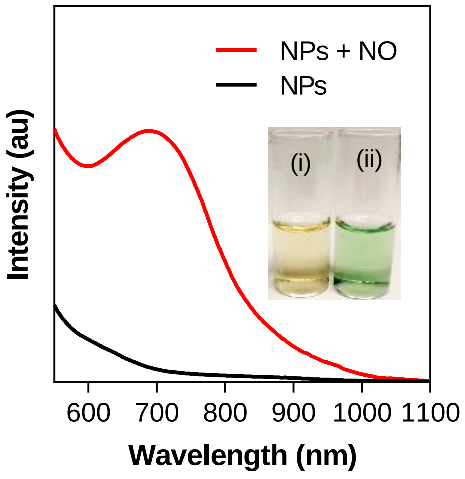

Supplementary Fig. 20 Absorption spectra of OTTAB NPs before and after the treatment of NO. Inset shows the photographs of OTTAB NPs solution (i) without and (ii) with the treatment of NO.

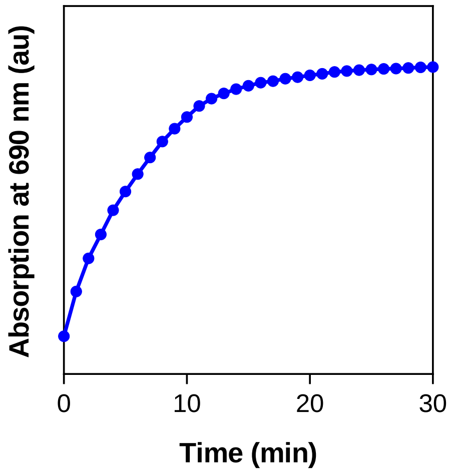

Supplementary Fig. 21 Absorption of OTTAB NPs at 690 nm upon reaction with NO for different time.

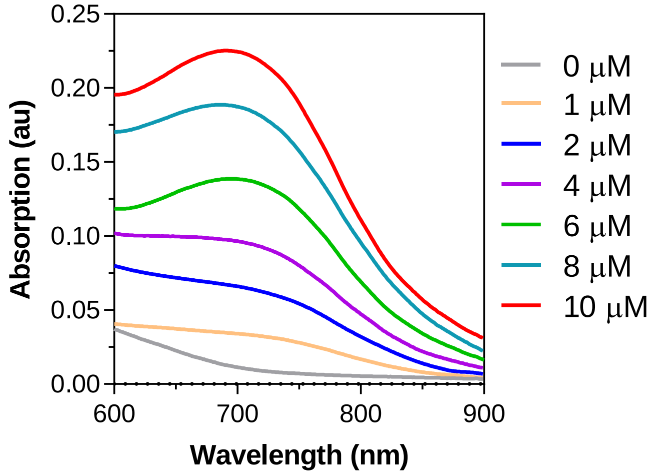

Supplementary Fig. 22 Absorption spectra of OTTAB NPs with the treatment of different concentrations of NO (0, 1, 2, 4, 6, 8, 10 μM).

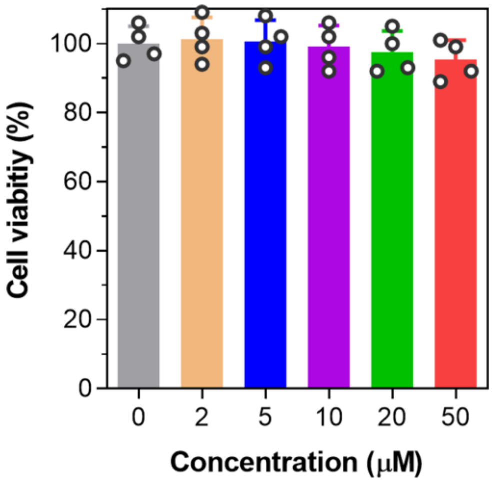

Supplementary Fig. 23 Cell viabilities of Detroit 551 cells treated with different concentrations of OTTAB NPs. Data are presented as mean ± s.d. (*n* = 4 independent experiments).

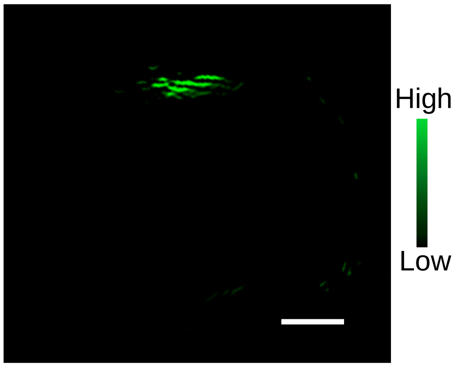

Supplementary Fig. 24 Representative noninvasive in vivo PA image of the whole mouse brain after intracerebroventricularly injecting OTTAB NPs. Scale bar: 3 mm.

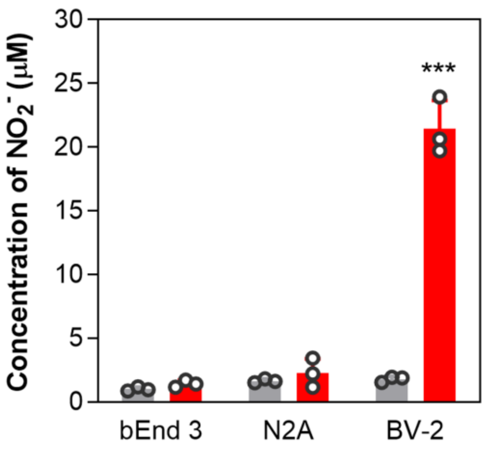

Supplementary Fig. 25 The NO_2^- concentration generated from bEnd 3, N2A and BV-2 cells after the same LPS stimulation. The bEnd 3 (endothelial cell), N2A (neuron) and BV-2 cells (microglia) were used to investigate the NO production after LPS stimulation.[1,2] The NO generation from BV-2 cell is very high, and the other two can be negligible. Grey: without LPS treatment, red: with LPS treatment. Data are presented as mean ± s.d. ($n = 3$ independent experiments). ***$p = 0.00011$ in comparison between the NO_2^- concentration of BV-2 cells without and with LPS treatment using two-tailed unpaired *t*-test.

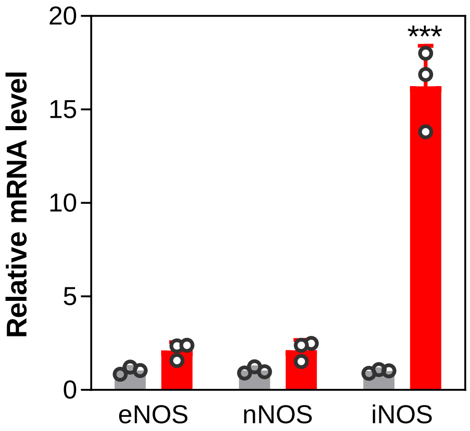

Supplementary Fig. 26 Relative mRNA levels of eNOS, nNOS and iNOS at the disease site in the LPS-induced encephalitis model. Grey: without LPS treatment, red: with LPS treatment. Data are presented as mean ± s.d. ($n = 3$ biologically independent mice per group). ***$p = 0.00026$ in comparison between the relative mRNA level of iNOS without and with LPS treatment using two-tailed unpaired *t*-test.

a b

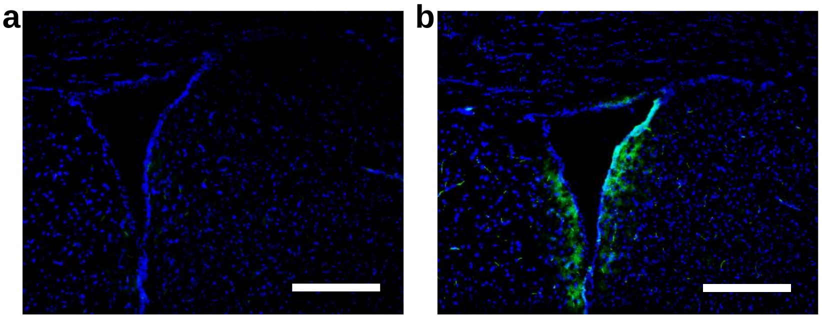

Supplementary Fig. 27 Immunofluorescent staining of iNOS in **a** normal and **b** inflamed mouse brain. Nuclei were stained with DAPI (blue signal), and iNOS was stained with the anti-iNOS primary antibody followed by FITC-labeled secondary antibody (green signal). Scale bars: 250 μm. Brain tissues were harvested from three mice in each group, and the representative images from each group are shown.

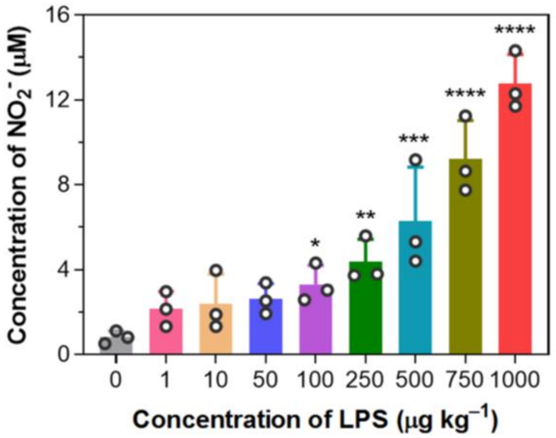

Supplementary Fig. 28 The NO_2^- concentration at the disease site versus the concentration of LPS injected into mouse brain. Data are presented as mean ± SD (*n* = 3 biologically independent mice). Data are presented as mean ± s.d. (*n* = 3 biologically independent mice per group). **p* = 0.038, ***p* = 0.0051, ****p* = 0.00011, *****p* < 0.0001 (750 vs. 0: $p = 5.7 \times 10^{-7}$, 1000 vs. 0: $p = 3 \times 10^{-9}$) compared to 0 μg kg^{-1} of LPS with one-way ANOVA for multiple comparisons.

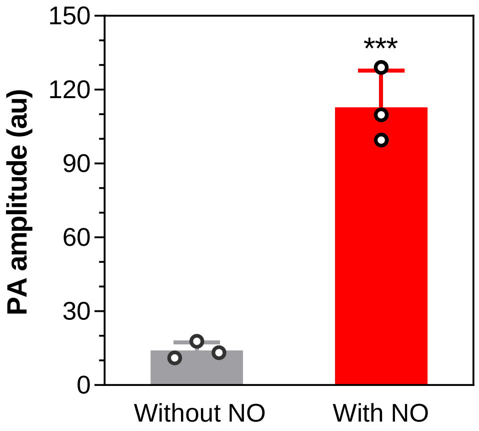

Supplementary Fig. 29 PA amplitudes of OTTAB@NEs without and with the treatment of NO. Data are presented as mean ± s.d. ($n = 3$ independent experiments). ***p = 0.00037 using two-tailed unpaired t-test.

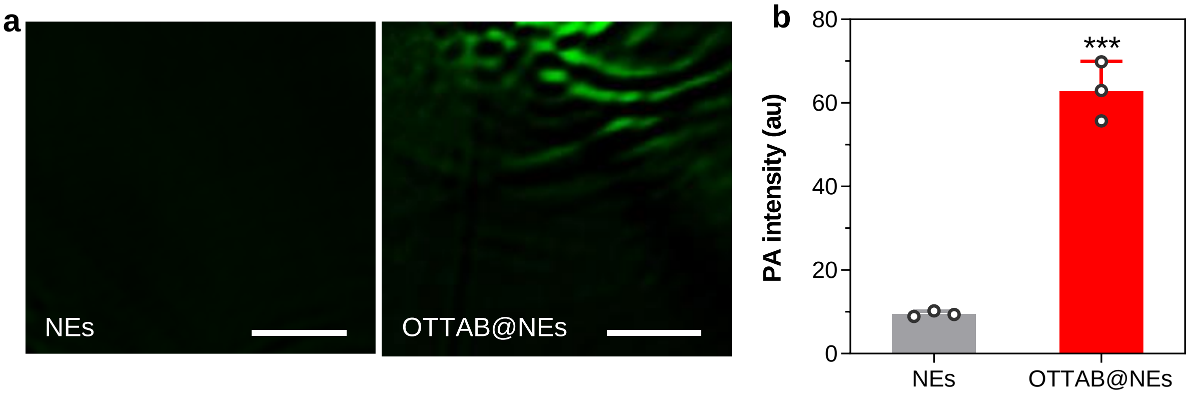

Supplementary Fig. 30 **a** Representative in vivo noninvasive PA images and **b** the corresponding PA intensity of LPS-induced encephalitis after intravenous administration of NEs and OTTAB@NEs. Scale bars: 1 mm. Data are presented as mean ± s.d. ($n = 3$ biologically independent mice per group). ***p = 0.0002 using two-tailed unpaired t-test.

Supplementary Table 1. Cartesian coordinates of OTTAB calculated by the DFT, B3LYP/6-31G(d), Gaussian 09 program.

atom	x	y	z
C	-7.0887	-0.75667	-0.29863
C	-8.02601	-1.79427	-0.46535
C	-9.39166	-1.56302	-0.37921
C	-9.88969	-0.27677	-0.09612
C	-8.95965	0.76298	0.08906
C	-7.59612	0.52607	-0.02119
N	-11.27644	-0.03591	-0.00121
C	-11.82348	1.21194	-0.42235
C	-12.15752	-1.04424	0.48754
C	-13.3516	-1.34247	-0.19123
C	-14.21564	-2.31659	0.28826
C	-13.90209	-3.03811	1.44974
C	-12.71414	-2.75116	2.13232
C	-11.86169	-1.75285	1.65715
C	-12.75355	1.89325	0.38115
C	-13.29796	3.10079	-0.03307
C	-12.91327	3.67406	-1.25442
C	-11.98431	3.00534	-2.06063
C	-11.45892	1.7798	-1.64775
C	12.62361	1.92982	-0.19411
C	13.53373	2.64237	0.573
C	13.38524	2.71896	1.96612
C	12.31317	2.05804	2.57674
C	11.41463	1.3236	1.80003
C	10.63593	-0.4419	-2.65676
C	11.14526	-1.328	-3.59496
C	12.19782	-2.1934	-3.25947
C	12.72295	-2.16053	-1.96263
C	12.19002	-1.28147	-1.01768
C	11.14762	-0.40982	-1.34751
C	11.54847	1.25463	0.4092
N	10.63045	0.50521	-0.38357
C	7.30249	1.95776	0.43196
C	8.66957	1.8455	0.22446
C	9.24056	0.62208	-0.17669
C	8.38099	-0.47526	-0.36778
C	7.01349	-0.35152	-0.15976
C	6.43481	0.86315	0.25106
C	1.09428	0.26868	0.33813

C	0.61967	-0.25927	-0.91034
C	-0.75261	-0.71365	-1.0741
C	-1.70855	-0.59233	-0.00414
C	-1.21626	-0.11272	1.20394
C	0.17545	0.27458	1.38583
N	1.36199	-0.40679	-2.00729
S	0.38766	-1.07549	-3.15888
N	-1.01134	-1.19118	-2.29143
N	-1.99059	-0.04089	2.36756
N	0.5088	0.66474	2.68171
C	4.99737	0.99688	0.49471
S	3.83486	-0.05472	-0.28712
C	2.47448	0.72493	0.50969
C	2.93737	1.76388	1.29891
C	4.34609	1.91607	1.28984
C	-3.11069	-0.95849	-0.23604
S	-4.46334	0.02415	0.34187
C	-5.65391	-1.01941	-0.41896
C	-5.01386	-2.04402	-1.07914
C	-3.59874	-2.00694	-0.98175
O	-13.49378	4.87141	-1.56237
C	-14.99147	13.17855	-7.07589
C	-14.27367	11.83086	-6.9561
C	-14.62792	11.06729	-5.67427
C	-13.90935	9.71831	-5.54773
C	-14.25947	8.95092	-4.26736
C	-13.5302	7.60643	-4.15017
C	-13.87177	6.83542	-2.86958
C	-13.12336	5.51282	-2.78127
O	-14.80927	-3.98708	1.82576
C	-18.375	-11.81267	7.05665
C	-17.25179	-10.78082	6.91765
C	-17.47093	-9.79898	5.76
C	-16.34925	-8.76395	5.61208
C	-16.56467	-7.79015	4.44726
C	-15.4388	-6.75975	4.29525
C	-15.65227	-5.80586	3.11369
C	-14.52287	-4.79627	2.96477
O	12.63561	-3.01613	-4.25803
C	18.24423	-10.30731	-8.37301
C	17.92402	-9.49505	-7.11472
C	16.83052	-8.44237	-7.33433
C	16.50514	-7.62657	-6.0773

C	15.4066	-6.57829	-6.29108
C	15.08824	-5.76722	-5.02876
C	13.97998	-4.72804	-5.23736
C	13.68763	-3.93508	-3.97136
O	14.32847	3.45409	2.62606
C	18.95661	7.61493	10.53997
C	17.78493	6.75477	10.05737
C	17.76939	6.54935	8.53767
C	16.59895	5.68909	8.0464
C	16.58377	5.48533	6.5267
C	15.41325	4.62355	6.03721
C	15.4046	4.42698	4.5165
C	14.24041	3.56728	4.0447
H	-7.67767	-2.8034	-0.66188
H	-10.08473	-2.38419	-0.5236
H	-9.31449	1.76314	0.3109
H	-6.91068	1.35939	0.10508
H	-13.59774	-0.80174	-1.09916
H	-15.13788	-2.55039	-0.23339
H	-12.44835	-3.28169	3.0387
H	-10.94901	-1.52821	2.19948
H	-13.04863	1.46475	1.33335
H	-14.01632	3.63031	0.58406
H	-11.67409	3.41575	-3.01377
H	-10.74924	1.26171	-2.28441
H	12.7413	1.88573	-1.27187
H	14.36604	3.16372	0.11172
H	12.17462	2.09209	3.65062
H	10.59456	0.80185	2.28245
H	9.83123	0.23228	-2.93131
H	10.75246	-1.3575	-4.60594
H	13.53337	-2.81772	-1.6717
H	12.59555	-1.26834	-0.01134
H	6.89235	2.92451	0.70742
H	9.30656	2.71291	0.35712
H	8.79394	-1.43208	-0.66675
H	6.38498	-1.22817	-0.28796
H	-2.95317	-0.33042	2.25429
H	-1.92284	0.84697	2.85334
H	1.50793	0.68831	2.85079
H	0.01029	0.12452	3.38171
H	2.27314	2.42523	1.84357
H	4.86551	2.66975	1.87019

H	-5.54717	-2.79822	-1.64617
H	-2.94551	-2.72036	-1.46616
H	-14.71829	13.69784	-8.0002
H	-16.07981	13.04933	-7.07658
H	-14.73914	13.83774	-6.23748
H	-13.18738	11.99003	-6.99307
H	-14.51748	11.20719	-7.82691
H	-15.71488	10.90597	-5.63755
H	-14.38586	11.69172	-4.80234
H	-12.82285	9.88191	-5.58564
H	-14.15126	9.09551	-6.42078
H	-15.34456	8.78065	-4.22903
H	-14.01874	9.5716	-3.39294
H	-12.44566	7.78057	-4.19292
H	-13.77238	6.98748	-5.02569
H	-14.9492	6.6356	-2.8229
H	-13.62494	7.43857	-1.98739
H	-12.03668	5.68265	-2.8002
H	-13.37478	4.86866	-3.63689
H	-18.18839	-12.49864	7.88924
H	-19.34042	-11.32627	7.23685
H	-18.47477	-12.4144	6.14613
H	-16.29406	-11.29954	6.77466
H	-17.15343	-10.21776	7.85577
H	-18.42922	-9.27923	5.90258
H	-17.57044	-10.36293	4.82144
H	-15.39095	-9.2852	5.47564
H	-16.25357	-8.19593	6.54847
H	-17.52065	-7.26521	4.58288
H	-16.66172	-8.35886	3.51181
H	-14.48197	-7.28661	4.1718
H	-15.34929	-6.18107	5.22549
H	-16.59713	-5.26166	3.23246
H	-15.73396	-6.37588	2.1802
H	-13.55953	-5.30946	2.82775
H	-14.44133	-4.16816	3.86428
H	19.02572	-11.05013	-8.18282
H	17.35881	-10.84188	-8.73548
H	18.59249	-9.65931	-9.18535
H	18.83655	-8.99901	-6.75693
H	17.61393	-10.17469	-6.30928
H	15.91712	-8.93865	-7.69239
H	17.13968	-7.76153	-8.14033

H	17.41841	-7.12785	-5.72244
H	16.20148	-8.30946	-5.271
H	14.49184	-7.07587	-6.64244
H	15.70737	-5.89334	-7.09616
H	16.00261	-5.26445	-4.68302
H	14.79694	-6.45511	-4.22237
H	13.05621	-5.22118	-5.56351
H	14.26133	-4.02883	-6.03415
H	14.58412	-3.39002	-3.6404
H	13.38639	-4.60776	-3.15462
H	18.93919	7.74251	11.6272
H	19.91786	7.16053	10.27416
H	18.92827	8.61286	10.08785
H	16.83851	7.21732	10.36899
H	17.82053	5.77558	10.55411
H	18.71672	6.08675	8.22547
H	17.73411	7.52935	8.04048
H	15.65217	6.15183	8.35976
H	16.63534	4.70921	8.54363
H	17.52983	5.02272	6.21218
H	16.54533	6.4643	6.02864
H	14.46723	5.08584	6.35296
H	15.45334	3.64384	6.53415
H	16.34073	3.95683	4.19138
H	15.34969	5.39862	4.01057
H	13.28042	4.02436	4.32747
H	14.28341	2.56942	4.50592

Supplementary Table 2. Cartesian coordinates of OTTTB calculated by the DFT, B3LYP/6-31G(d), Gaussian 09 program.

atom	x	y	z
C	6.96397	-0.93346	0.20347
C	7.5143	-0.3721	-0.96463
C	8.86949	-0.09313	-1.07068
C	9.74667	-0.3607	-0.00266
C	9.20528	-0.91948	1.17159
C	7.84961	-1.19603	1.26741
N	11.12276	-0.07549	-0.10174
C	12.08301	-0.89924	0.55669
C	11.58324	1.03451	-0.86941
C	12.66208	0.8894	-1.75837
C	13.12878	1.97221	-2.48955
C	12.51758	3.22932	-2.36976
C	11.43595	3.38283	-1.49428
C	10.98731	2.29419	-0.74416
C	13.11767	-0.32435	1.31425
C	14.06803	-1.12168	1.9355
C	14.00147	-2.51953	1.83395
C	12.96872	-3.10296	1.09033
C	12.02919	-2.29303	0.44973
C	-12.10145	-0.45862	2.30829
C	-12.56331	0.27141	3.39404
C	-12.15951	1.60217	3.58154
C	-11.29696	2.19262	2.65028
C	-10.85442	1.45848	1.54854
C	-11.46975	-2.69658	-0.88497
C	-12.39256	-3.41461	-1.631
C	-13.60385	-2.82488	-2.02417
C	-13.86943	-1.49937	-1.66136
C	-12.92585	-0.77614	-0.92879
C	-11.71988	-1.35971	-0.52851
C	-11.23725	0.12547	1.36659
N	-10.77379	-0.62362	0.24441
C	-7.06677	-0.47297	0.51584
C	-8.4072	-0.49769	0.87019
C	-9.41136	-0.61136	-0.11099
C	-9.00988	-0.71318	-1.45762
C	-7.66625	-0.70051	-1.80077
C	-6.6545	-0.572	-0.828
C	-1.31336	-0.76931	-0.96516
C	-0.25451	-0.49134	-1.90706
C	1.17109	-0.65043	-1.5705
C	1.6307	-1.11003	-0.29505
C	0.57174	-1.32836	0.58832

C	-0.81362	-1.1742	0.28346
N	-0.43612	-0.05618	-3.16066
S	1.04121	0.13578	-3.83761
N	1.98632	-0.32524	-2.58181
C	-5.24641	-0.5381	-1.21158
S	-3.97938	-0.91851	-0.06737
C	-2.71551	-0.62932	-1.26884
C	-3.29982	-0.27326	-2.48112
C	-4.7045	-0.22495	-2.44765
C	3.02013	-1.32626	0.05944
S	4.34618	-0.44783	-0.68709
C	5.53937	-1.23588	0.32639
C	4.92316	-2.13169	1.17822
C	3.51851	-2.18205	1.0305
N	0.5466	-1.70193	1.90552
N	-0.72895	-1.74983	2.3859
N	-1.53685	-1.45713	1.43039
O	14.97914	-3.21174	2.48896
C	20.18028	-11.33373	5.39064
C	18.98222	-10.80126	4.59903
C	18.84171	-9.27545	4.66507
C	17.64427	-8.73566	3.87387
C	17.50672	-7.20955	3.93349
C	16.30829	-6.67463	3.13979
C	16.1839	-5.14698	3.18923
C	14.98997	-4.63507	2.39597
O	13.04699	4.22485	-3.1395
C	14.02473	13.52816	-6.86092
C	13.22669	12.60495	-5.93547
C	13.75868	11.16683	-5.9088
C	12.96708	10.23598	-4.98234
C	13.49991	8.79839	-4.9577
C	12.71301	7.87038	-4.02382
C	13.24949	6.43395	-4.01031
C	12.47156	5.52801	-3.06644
O	-14.44544	-3.62102	-2.74628
C	-22.70578	-6.06102	-7.95443
C	-21.97246	-4.9671	-7.17268
C	-20.61498	-5.42222	-6.62293
C	-19.87263	-4.3328	-5.83987
C	-18.51536	-4.79029	-5.29216
C	-17.77253	-3.70083	-4.50894
C	-16.41522	-4.16813	-3.96963
C	-15.68769	-3.08041	-3.1923
O	-12.65641	2.22656	4.68945
C	-13.58425	9.71452	11.37141

C	-12.97621	9.25115	10.0443
C	-13.36525	7.81649	9.66706
C	-12.75721	7.3462	8.34023
C	-13.14119	5.91084	7.96143
C	-12.52106	5.44647	6.63771
C	-12.89814	4.00889	6.26011
C	-12.25344	3.56929	4.95324
H	6.87394	-0.17978	-1.82082
H	9.26192	0.32408	-1.99121
H	9.85569	-1.11827	2.01608
H	7.46141	-1.59173	2.20089
H	13.13558	-0.08077	-1.86714
H	13.96313	1.86532	-3.17488
H	10.94639	4.34171	-1.37415
H	10.15838	2.42582	-0.05641
H	13.17315	0.75539	1.40574
H	14.87073	-0.68279	2.51885
H	12.89298	-4.17869	0.98806
H	11.24075	-2.75189	-0.13794
H	-12.40908	-1.49119	2.17989
H	-13.22882	-0.17554	4.12519
H	-10.9749	3.22089	2.76039
H	-10.19575	1.92705	0.82461
H	-10.54274	-3.16689	-0.57377
H	-12.20417	-4.446	-1.91021
H	-14.79496	-1.01643	-1.95076
H	-13.133	0.25407	-0.65829
H	-6.32236	-0.36801	1.29957
H	-8.68792	-0.4239	1.91467
H	-9.76136	-0.80521	-2.23363
H	-7.39391	-0.79957	-2.84675
H	-2.70728	-0.04552	-3.35523
H	-5.30604	0.06394	-3.30147
H	5.47139	-2.7707	1.86039
H	2.89685	-2.89047	1.56658
H	1.32131	-1.79446	2.54405
H	20.25305	-12.42403	5.32301
H	21.12089	-10.91409	5.01606
H	20.10258	-11.07113	6.45185
H	18.05989	-11.26551	4.97396
H	19.07062	-11.11021	3.54859
H	19.76475	-8.81048	4.28996
H	18.75333	-8.96529	5.71617
H	16.7214	-9.19846	4.25185
H	17.73176	-9.05031	2.82408
H	18.42813	-6.74618	3.55369

H	17.41814	-6.8923	4.982
H	15.38609	-7.13159	3.52597
H	16.39368	-7.00085	2.09353
H	17.09366	-4.68158	2.79077
H	16.08727	-4.80866	4.22805
H	14.05202	-5.04621	2.79776
H	15.06461	-4.94201	1.34222
H	13.62209	14.54625	-6.85627
H	15.07534	13.58485	-6.5541
H	14.00335	13.16709	-7.89548
H	12.1735	12.59506	-6.24759
H	13.23567	13.0125	-4.91536
H	14.81341	11.17703	-5.59845
H	13.74876	10.75826	-6.92952
H	11.91229	10.22692	-5.29209
H	12.97862	10.64491	-3.96184
H	14.55579	8.80683	-4.6528
H	13.48249	8.38622	-5.9764
H	11.65608	7.86482	-4.32559
H	12.73563	8.28085	-3.0043
H	14.30439	6.42778	-3.70987
H	13.20841	6.00606	-5.0194
H	11.41001	5.48696	-3.35224
H	12.5249	5.90628	-2.03486
H	-23.66937	-5.70566	-8.33369
H	-22.11443	-6.39808	-8.81338
H	-22.89909	-6.93664	-7.3243
H	-22.60326	-4.6258	-6.34056
H	-21.82519	-4.09114	-7.81907
H	-19.98419	-5.76518	-7.4556
H	-20.76248	-6.29868	-5.97569
H	-20.50352	-3.99053	-5.00709
H	-19.72582	-3.45659	-6.48753
H	-17.88386	-5.13169	-6.12437
H	-18.6611	-5.66592	-4.64411
H	-18.40272	-3.36165	-3.67458
H	-17.62823	-2.8244	-5.15656
H	-15.77339	-4.49557	-4.79662
H	-16.54867	-5.03702	-3.31368
H	-16.28702	-2.75176	-2.33031
H	-15.50816	-2.20127	-3.82875
H	-13.28691	10.74032	11.61214
H	-14.67931	9.68533	11.3376
H	-13.26353	9.07252	12.19969
H	-11.88153	9.32614	10.0971
H	-13.28735	9.93301	9.24113

H	-14.46082	7.74101	9.61307
H	-13.05498	7.13396	10.47125
H	-11.66202	7.42468	8.39568
H	-13.06845	8.02903	7.53674
H	-14.23549	5.83067	7.89827
H	-12.83296	5.22716	8.76483
H	-11.42706	5.5316	6.70332
H	-12.83082	6.13003	5.8345
H	-13.98688	3.91486	6.16605
H	-12.59001	3.31648	7.05297
H	-11.15726	3.62405	5.02617
H	-12.56597	4.22527	4.12721

References:

1. Kacimi, R., Giffard, R. G. & Yenari, M. A. Endotoxin-activated microglia injure brain derived endothelial cells via NF-*κ*B, JAK-STAT and JNK stress kinase pathways. *J. Inflamm.* **8**, 7 (2011).

2. Kacimi, R. & Yenari, M. A. Pharmacologic heat shock protein 70 induction confers cytoprotection against inflammation in gliovascular cells. *Glia.* **63**, 1200–1212 (2015).
