# Supplementary material for: Facilitation of molecular motion to develop turn-on photoacoustic bioprobe for detecting nitric oxide in encephalitis
Source: Nat Commun. 2021 Feb 11;12:960. doi: 10.1038/s41467-021-21208-1 (PMC7878857; doi:10.1038/s41467-021-21208-1)
Supplement: Supplementary file 3 — Reporting Summary [file 41467_2021_21208_MOESM3_ESM.pdf]

nature research

Corresponding author(s): Ben Zhong Tang, Xue Xue

Last updated by author(s): Dec 10, 2020

Reporting Summary

Nature Research wishes to improve the reproducibility of the work that we publish. This form provides structure for consistency and transparency in reporting. For further information on Nature Research policies, see our Editorial Policies and the Editorial Policy Checklist.

Statistics

For all statistical analyses, confirm that the following items are present in the figure legend, table legend, main text, or Methods section.

n/a	Confirmed	
☐	☒	The exact sample size (*n*) for each experimental group/condition, given as a discrete number and unit of measurement
☐	☒	A statement on whether measurements were taken from distinct samples or whether the same sample was measured repeatedly
☐	☒	The statistical test(s) used AND whether they are one- or two-sided<br>*Only common tests should be described solely by name; describe more complex techniques in the Methods section.*
☒	☐	A description of all covariates tested
☒	☐	A description of any assumptions or corrections, such as tests of normality and adjustment for multiple comparisons
☐	☒	A full description of the statistical parameters including central tendency (e.g. means) or other basic estimates (e.g. regression coefficient) AND variation (e.g. standard deviation) or associated estimates of uncertainty (e.g. confidence intervals)
☐	☒	For null hypothesis testing, the test statistic (e.g. *F*, *t*, *r*) with confidence intervals, effect sizes, degrees of freedom and *P* value noted<br>*Give P values as exact values whenever suitable.*
☒	☐	For Bayesian analysis, information on the choice of priors and Markov chain Monte Carlo settings
☒	☐	For hierarchical and complex designs, identification of the appropriate level for tests and full reporting of outcomes
☒	☐	Estimates of effect sizes (e.g. Cohen's *d*, Pearson's *r*), indicating how they were calculated

Our web collection on statistics for biologists contains articles on many of the points above.

Software and code

Policy information about availability of computer code

Data collection	SoftMax Pro 6 software (version 6.5.1) for platereader, and Real-Time PCR Detection Systems Bio-red CFXMaestro (version 1.0) for PCR sample information acquisition in this study.
Data analysis	View MSOT software suite (version 3.6) was used for PA image reconstruction. Gaussian 09 program package (revision D. 01) was used to get the optimized molecular geometry. All statistical analyses were carried out with GraphPad Prism software (version 8.0.2).

For manuscripts utilizing custom algorithms or software that are central to the research but not yet described in published literature, software must be made available to editors and reviewers. We strongly encourage code deposition in a community repository (e.g. GitHub). See the Nature Research guidelines for submitting code & software for further information.

Data

Policy information about availability of data

All manuscripts must include a data availability statement. This statement should provide the following information, where applicable:

- Accession codes, unique identifiers, or web links for publicly available datasets
- A list of figures that have associated raw data
- A description of any restrictions on data availability

All the data supporting the findings in this study are available in the paper and Supplementary information files. All the data related to this paper are available from the corresponding authors upon reasonable request. Source data are provided with this paper.

Field-specific reporting

Please select the one below that is the best fit for your research. If you are not sure, read the appropriate sections before making your selection.

☒ Life sciences ☐ Behavioural & social sciences ☐ Ecological, evolutionary & environmental sciences

For a reference copy of the document with all sections, see nature.com/documents/nr-reporting-summary-flat.pdf

Life sciences study design

All studies must disclose on these points even when the disclosure is negative.

Sample size	No sample size calculations were performed. In vitro studies were repeated three times independently with triplicate or quintuplicate samples, and in the in vivo experiments with 3 mice per group were performed. Statistics such as error bars, significance and p values can be derived from n ≥ 3.
Data exclusions	No data were excluded from the analyses.
Replication	All experiments were repeated three times independently and experimental findings were reproducible.
Randomization	All samples/organisms were randomly allocated into experimental groups.
Blinding	All the investigators were blinded to group allocation in the course of data collection and analysis.

Reporting for specific materials, systems and methods

We require information from authors about some types of materials, experimental systems and methods used in many studies. Here, indicate whether each material, system or method listed is relevant to your study. If you are not sure if a list item applies to your research, read the appropriate section before selecting a response.

Materials & experimental systems

n/a	Involved in the study	
☐	☒	Antibodies
☐	☒	Eukaryotic cell lines
☒	☐	Palaeontology and archaeology
☐	☒	Animals and other organisms
☒	☐	Human research participants
☒	☐	Clinical data
☒	☐	Dual use research of concern

Methods

n/a	Involved in the study	
☒	☐	ChIP-seq
☒	☐	Flow cytometry
☒	☐	MRI-based neuroimaging

Antibodies

Antibodies used	Anti-iNOS antibody (D6B6S, Cell Signaling Technology, Inc. Rabbit mAb, #13120S, dilution: 1:1000) and Fluorescein (FITC)-conjugated AffiniPure Goat anti-Rabbit IgG (H+L) (Jackson Immuno Research LABORATORIES, INC, 148563, dilution: 1:200).
Validation	Anti-iNOS antibody (D6B6S, Cell Signaling Technology, Inc. Rabbit mAb, #13120S). Specificity/Sensitivity provided by company, iNOS (D6B6S) Rabbit mAb recognizes endogenous levels of total iNOS protein. This antibody does not cross-react with other NOS proteins. The validation statements of anti-iNOS antibody can be found on the manufacturer's website: https://www.cellsignal.cn/products/primary-antibodies/inos-d6b6s-rabbit-mab/13120?N=4294956287&Ntt=inos&fromPage=plp.<br>Fluorescein (FITC)-conjugated AffiniPure Goat anti-Rabbit IgG (H+L) (Jackson Immuno Research LABORATORIES, INC, 148563). Specificity and Sensitivity provide by company. Based on immunoelectrophoresis and/or ELISA, the antibody reacts with whole molecule rabbit IgG. It also reacts with the light chains of other rabbit immunoglobulins. No antibody was detected against non-immunoglobulin serum proteins. The antibody may cross-react with immunoglobulins from other species.

Eukaryotic cell lines

Policy information about cell lines

Cell line source(s)	BV-2, N2A, bEnd 3 and Detroit 551 cell lines were purchased from ATCC. Primary neutrophils were freshlyisolated from 8 weeks mice in our lab according to the standard protocol as described in Methods section.
Authentication	Primary neutrophils were from our lab and freshly isolated from our animal colony. The other cells lines used were not authenticated in our lab.

Mycoplasma contamination	Cells were routinely screened for free of Mycoplasma contaminations. All cell lines are Mycoplasma negative with this study.
Commonly misidentified lines (See ICLAC register)	None of the cell lines used in this study is commonly misidentified cell line.

Animals and other organisms

Policy information about studies involving animals; ARRIVE guidelines recommended for reporting animal research

Laboratory animals	The Kunming mice (6-8 weeks) were purchased from SPF (Beijing) Biotechnology Co., Ltd.
Wild animals	The study did not involve wild animals.
Field-collected samples	The study did not involve samples collected from the filed.
Ethics oversight	All animal studies were conducted under the guidelines set by Tianjin Committee of Use and Care of Laboratory Animals, and the overall project protocols were approved by the Animal Ethics Committee of Nankai University.

Note that full information on the approval of the study protocol must also be provided in the manuscript.
